# Anti-Osteoclastogenic and Antibacterial Effects of Chlorinated Polyketides from the Beibu Gulf Coral-Derived Fungus *Aspergillus unguis* GXIMD 02505

**DOI:** 10.3390/md20030178

**Published:** 2022-02-28

**Authors:** Yanting Zhang, Zhichao Li, Bingyao Huang, Kai Liu, Shuai Peng, Xinming Liu, Chenghai Gao, Yonghong Liu, Yanhui Tan, Xiaowei Luo

**Affiliations:** 1 Institute of Marine Drugs, Guangxi University of Chinese Medicine, Nanning 530200, China; ting9097@163.com (Y.Z.); hby03502@126.com (B.H.); kailiu@gxtcmu.edu.cn (K.L.); pengshuai0208@163.com (S.P.); leosimon0917@gmail.com (X.L.); gaoch@gxtcmu.edu.cn (C.G.); 2 State Key Laboratory for Chemistry and Molecular Engineering of Medicinal Resources, School of Chemistry and Pharmaceutical Sciences, Guangxi Normal University, Guilin 541004, China; lizhichao3278@163.com

**Keywords:** marine fungi, *Aspergillus unguis*, depsides, depsidones, osteoclast differentiation, antibacterial

## Abstract

One new depsidone derivative, aspergillusidone H (**3**), along with seven known biosynthetically related chlorinated polyketides, were obtained from the Beibu Gulf coral-derived fungus *Aspergillus unguis* GXIMD 02505. Their structures were determined by comprehensive physicochemical and spectroscopic data interpretation. Notably, the X-ray crystal structure of **2** and the previously unknown absolute configuration of **8,** assigned by ECD calculations, are described here for the first time. Compounds **1**–**5**, **7** and **8** exhibited inhibition of lipopolysaccharide (LPS)-induced NF-κB in RAW 264.7 macrophages at 20 μM. In addition, the two potent inhibitors (**2** and **7**) dose-dependently suppressed RANKL-induced osteoclast differentiation without any evidence of cytotoxicity in bone marrow macrophages cells (BMMs). This is the first report of osteoclastogenesis inhibitory activity for the metabolites of these kinds. Besides, compounds **1**, **2**, **4**, and **6**–**8** showed inhibitory activity against marine biofilm-forming bacteria, methicillin-resistant *Staphylococcus aureus*, *Microbulbifer variabilis*, *Marinobacterium jannaschii*, and *Vibrio pelagius*, with their MIC values ranging from 2 to 64 μg/mL. These findings provide a basis for further development of chlorinated polyketides as potential inhibitors of osteoclast differentiation and/or for use as anti-fouling agents.

## 1. Introduction

Bone is a rigid, yet dynamic, organ that provides maximal strength with minimal mass, supporting the human body and producing indispensable red and white blood cells [1]. Normal bone homeostasis is known to be regulated and maintained by two bone remodeling metabolic processes, bone resorption by osteoclasts and bone formation by osteoblasts [2]. With an ageing population, osteoporosis, particularly postmenopausal osteoporosis, will pose severe worldwide concern to public health and economic development [3]. Thus, the development of therapeutic agents to treat osteoporosis is urgently needed. The differentiation of the osteoclast is modulated by two critical factors, the macrophage colony stimulation factor (M-CSF) and the receptor activator of the nuclear factor kappa-B (NF-κB) ligand (RANKL). RANKL signaling pathways have been recently proposed as key targets for inhibiting osteoclast differentiation and bone resorption [4]. Hence, many efforts have been recently devoted to the discovery of lead compounds from natural sources that target RANKL-induced osteoclast differentiation for attenuating enhanced bone resorption and bone loss [5]. 

Depsides, depsidones, and diphenyl ethers are biosynthetically-related aromatic polyketides originated normally from the condensation of orsellinic acid, orcinol, and/or polyphenolic units by ether and/or ester bonds [6], which are widely encountered in lichens, as well as in some fungi (especially in *Aspergillus unguis*) and higher plants [7]. Their variable linked patterns and different substituents lead to rich structural diversity. Naturally occurring depsides and depsidones were recently found with a wide array of remarkable bioactivities, including antimicrobial [8,9], α-glucosidase inhibitory [10,11], anti-inflammatory [12,13], cytotoxic [12], antioxidant [14], and anti-viral activities [15], arousing considerable interest from the communities of chemistry and pharmacology [6,16]. Curiously, a depsidone natural product, norstictic acid, was very recently reported as a key selective allosteric transcriptional regulator in a patient-derived model of triple-negative breast cancer [17]. 

**Figure 1:**
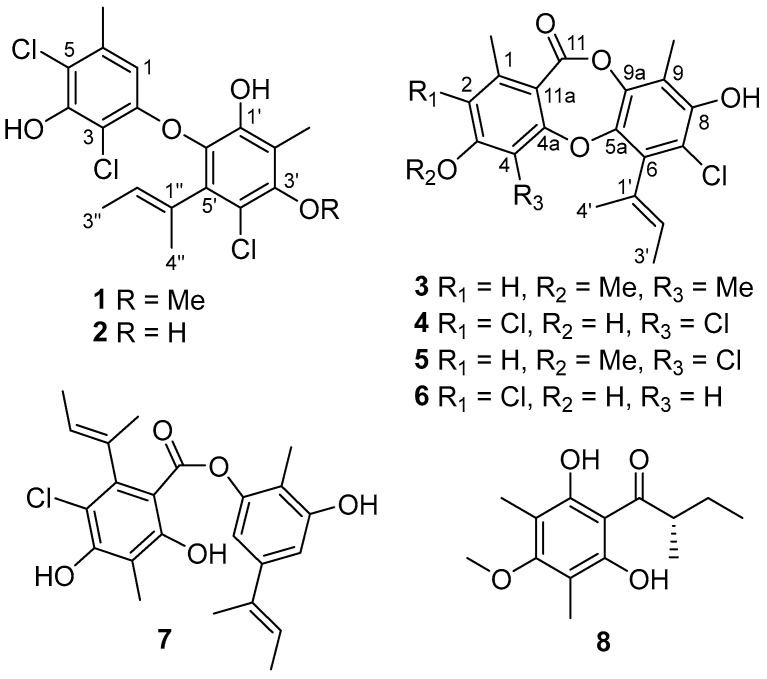
Structures of compounds **1**–**8**.

In our continuing endeavor to discover biologically active compounds from marine fungi, a series of structurally novel secondary metabolites (SM) with promising pharmacological effects have been recently discovered, including anti-tumor ascochlorins [18,19], chloroazaphilones [20] and diketopiperazine alkaloids [21], as well as nitrobenzoyl sesquiterpenoids as novel inhibitors of osteoclast differentiation [4]. In this study, a coral-derived fungus *Aspergillus unguis* GXIMD 02505 has drawn our attention, due to the intriguing characteristics of abundant chlorinated SMs as observed by the HPLC-UV/MS profile of its extract. Subsequent chemical investigation led to the isolation of eight diverse chlorinated aromatic polyketides (Figure 1) guided by the HPLC-UV/MS method. Several of them have displayed inhibitory effects on osteoclastogenesis by suppressing RANKL-induced NF-κB activation and on marine biofilm-forming bacteria. Herein, the isolation and structural determination, as well as their inhibitory activities on RANKL-induced osteoclastogenesis and antibacterial effects, are described in detail.

## 2. Results and Discussion

The cultures of *A. unguis* GXIMD 02505, based on rice fermentation, were extracted with EtOAc three times. The whole extract was then performed on the repeated column chromatography involving silica gel, reversed-phase silica gel C18, and semipreparative HPLC. The HPLC-DAD-guided purification resulted in the discovery of eight chlorinated aromatic polyketides, including two diphenyl ethers (**1** and **2**), four depsidones (**3**–**6**), one depside (**7**), and one related block (**8**). These obtained known compounds were identified as aspergillusethers J (**1**) [22] and F (**2**) [8], nornidulin (**4**) [8,23], aspergillusidones B and C (**5** and **6**) [23], guisinol (**7**) [24], and 1-(2,6-dihydroxy-4-methoxy-3,5-dimethylphenyl)-2-methylbutan-1-one (**8**) [25], respectively, by comparing their physicochemical and spectroscopic data with those reported. Notably, the X-ray crystal structure of **2** is described herein for the first time (Figure S19).

**Figure 2:**
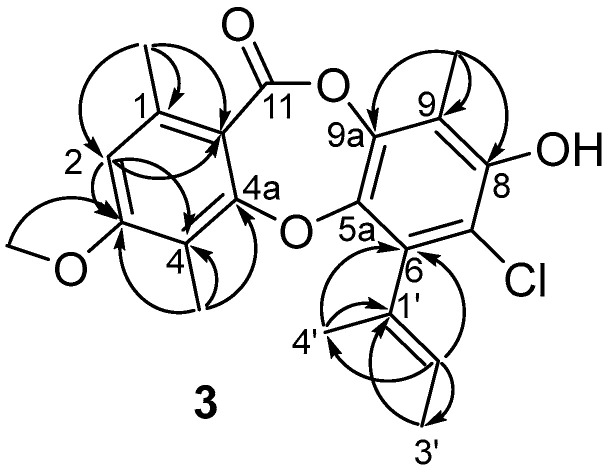
Key HMBC correlations for compound **3**.

**Figure 3:**
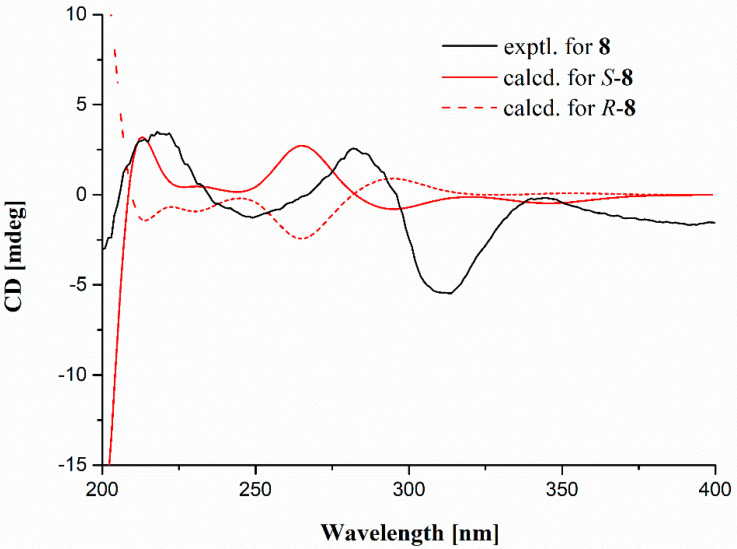
The experimental and calculated ECD spectra of **8**.

Compound **3** was obtained as white amorphous solid with the molecular formula C_21_H_21_ClO_5,_ as determined by a cluster of ion peaks at *m/z* 389.1159/391.1134 ([M + H]^+^) with a ratio of 3:1 in the HR-ESI-MS spectrum, indicative of a monochlorinated compound. The IR spectrum showed hydroxy and double-bond absorption bands at 3446 and 1653 cm^−1^, respectively. The UV spectrum exhibited absorption bands at 207 and 270 nm, indicating the presence of a benzene chromophore [8]. The ^1^H NMR data (Table 1) along with the HSQC experiment of **3** displayed the signals of two aromatic or olefinic protons, assigned to H-2 (*δ*_H_ 6.73, s) and H-2′ (*δ*_H_ 5.40, q, *J* = 6.8 Hz), five methyls, 1-Me (*δ*_H_ 2.45), 4-Me (*δ*_H_ 2.15), 9-Me (*δ*_H_ 2.24), H_3_-3′ (*δ*_H_ 1.82, d, *J* = 6.8 Hz), and H_3_-4′ (*δ*_H_ 1.91, s), and one methoxyl, 3-OMe (*δ*_H_ 3.87). Besides the above eight corresponding hydrogen-bearing carbons, twelve aromatic or olefinic (five oxygenated) ones and a carbonyl (*δ*_C_ 165.3) remained in the ^13^C NMR spectrum. The aforementioned NMR data of **3** highly resembled those of the co-isolation of a known compound, aspergillusidone B (**5**). A significant difference was the appearance of a methyl group (*δ*_H/C_ 2.15/10.2) at C-4 (*δ*_C_ 116.1) in **3** instead of a chlorine atom in **5**, which was also verified by the HMBC correlations (Figure 2) from 4-CH_3_ to C-3 (*δ*_C_ 163.0), C-4, and C-4a (*δ*_C_ 162.3). Consequently, **3** was assigned as aspergillusidone H. Besides, the previously unknown absolute configuration of **8** was determined by ECD calculations for the first time (Figure 3).

**Figure 4:**
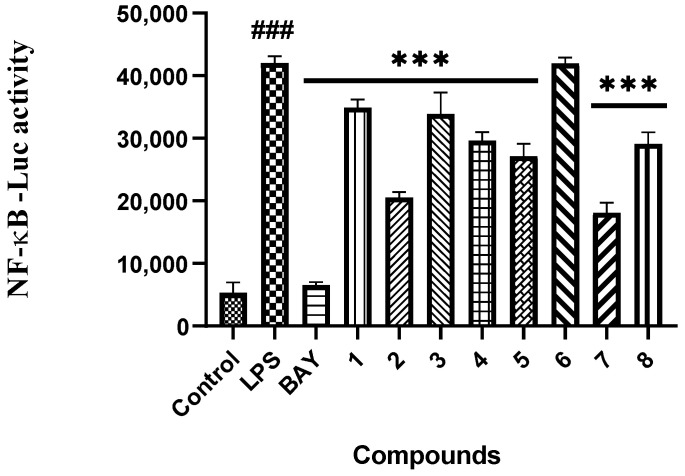
The inhibitory effects of compounds **1**–**8** on LPS-induced NF-κB activation in RAW264.7 cells at 20 μM. *n* = 3. ^###^ *p* < 0.001 vs. control group (untreated); *** *p* < 0.001 vs. LPS-induced group. BAY (BAY11-7082 treated, positive control).

**Figure 5:**
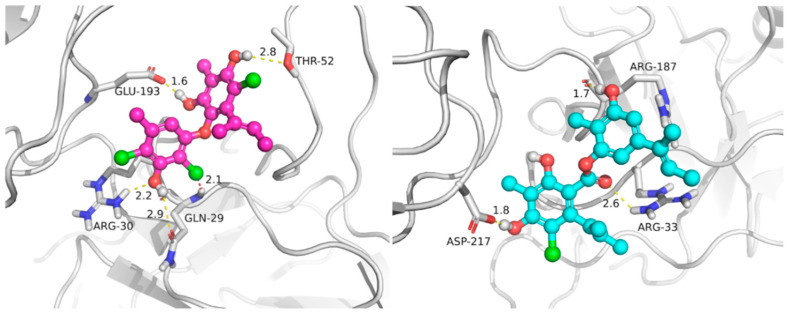
The predicted binding modes of compounds **2** (**left**) and **7** (**right**) with NF-κB p65 (PDB code: 3GUT, chain A) by molecular docking. The protein receptor is shown by cartoon and the highlighted interacting residues are shown by thick sticks. The yellow dashed lines represent hydrogen bonds. The red dashed line represents a halogen bond.

**Figure 6:**
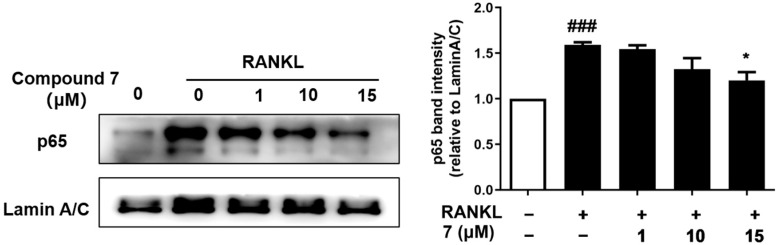
Compound **7** suppressed the RANKL-induced NF-κB p65 nuclear translocation in RAW264.7 cells. RAW264.7 cells were cultured with **7** (1, 10, or 15 μM) for 4 h, stimulated with RANKL (100 ng/mL) for 30 min, and then analyzed by Western blotting with p65 and lamin A/C. The relative nuclear protein expression levels of p65 to lamin A/C were determined using ImageJ software. *n* = 3, **^###^** *p* < 0.001 vs. untreated control, * *p* < 0.05 vs. RANKL-treated control.

Marine natural products have been recently found as a vital source of inhibitors of RANKL-induced osteoclastogenesis [4,26]. All of the compounds were then evaluated for their inhibitory effects against RANKL induced osteoclastogenesis in RAW 264.7 macrophages using the luciferase reporter gene, and in bone marrow macrophage cells (BMMs) by tartate-resisant acid phosphatase (TRAP) assays. Compounds **1**−**5**, **7** and **8** exhibited inhibition of lipopolysaccharide (LPS)-induced NF-κB activation in RAW 264.7 macrophages at 20 μM (*p* < 0.001) (Figure 4). To further investigate the binding modes of the two potent inhibitors (**2** and **7**) with NF-κB p65, a molecular docking study was primarily carried out using the Schrödinger suits software. The theoretical binding modes of **2** and **7** with NF-κB p65 protein was shown in Figure 5 with the nearly identical glide scores of −3.391 and −3.076 kcal/mol, respectively, which were consistent with the above NF-κB luciferase activity. Detailed analysis showed that the three hydroxy groups in **2** interact tightly via hydrogen bonds with the surrounding amino acid residues, GLU193, THR52, ARG30, and GLN29, meanwhile, the chlorine atom at C-3 also interacts with GLN29 by a halogen bond. Likewise, there are mainly hydrogen bond interactions between the residues of ASP217, ARG33, ARG187, and the hydroxy and ester groups in **7**. Moreover, compound **7** could block RANKL-induced NF-κB p65 nuclear translocation, as demonstrated by preliminary Western bolting analysis (Figure 6).

**Figure 7:**
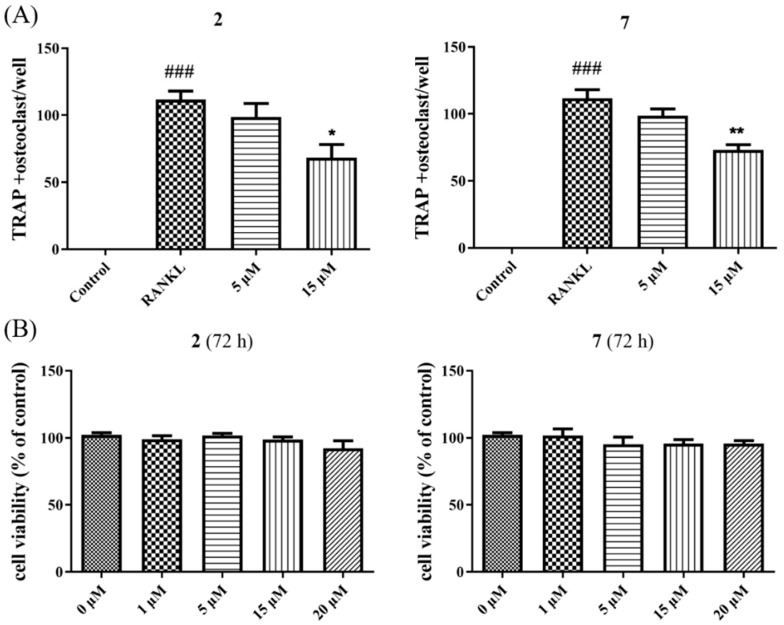
TRAP-positive multinucleated cells were regarded as quantified (**A**). Cell viability of **2** and **7** at different concentrations in BMMs for 72 h were measured by cell counting kit 8 assay (**B**). *n* = 3. ^###^ *p* < 0.001 vs. control group; * *p* < 0.05, ** *p* < 0.01 vs. RANKL group.

**Figure 8:**
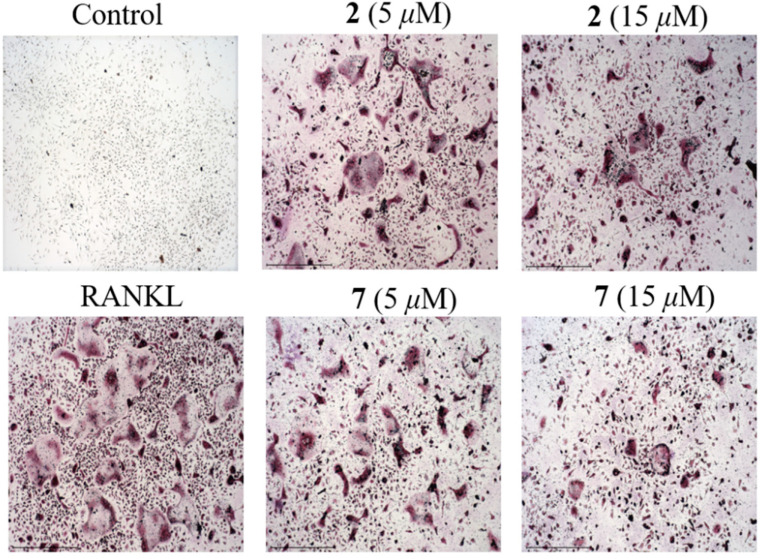
Representative images showing that RANKL induced osteoclast differentiation was inhibited by compounds **2** and **7** in a dose-dependent manner in BMMs. (magnification = 100×; scale bar = 500 μm).

Given that NF-κB plays a vital role in RANKL-induced osteoclast differentiation [4], the two potent inhibitors (**2** and **7**) were then further evaluated for the effects on RANKL-induced osteoclastogenesis in BMMs. Both of them dose-dependently suppressed RANKL-induced osteoclast differentiation without any evidence of cytotoxicity (Figure 7 and Figure 8). To our knowledge, the potent osteoclast differentiation inhibitory activity is revealed for these polyketides for the first time.

Marine biofouling, mainly accumulated by marine bacteria, algae, and invertebrates, adheres to the man-made surfaces of marine infrastructures, and is a thorny worldwide issue that causes huge losses in both marine technical and economic development [27]. Marine natural products (especially from marine microorganisms) have been recently evidenced as promising sources of antifouling lead compounds [28,29]. Thus, these isolated compounds were also evaluated for inhibitory activity against a series of marine biofilm-forming bacteria, including methicillin-resistant *Staphylococcus aureus* (MRSA), *Pseudomonas aeruginosa*, *Vibrio parahemolyticus*, *V. alginolyticus*, *V. tubiashii*, *Microbulbifer variabilis*, *Marinobacterium jannaschii*, *V. pelagius*, *V. rotiferianus*, and *Alteromonas macleodii*. Among them, compounds **2** and **4** showed significant activity against MRSA with the same MIC value of 2 μg/mL, as compared to that of the positive control ampicillin (1 μg/mL) (Table 2). Besides, compounds **1**, **6**, and **7** displayed moderate anti-MRSA activity with the MIC values ranging from 16 to 32 μg/mL. Compounds **1**, **2**, **4**, and **6**−**8** showed inhibition towards *M. variabilis*, *M. jannaschii*, and *V. pelagius*, with their MIC values ranging from 8 to 64 μg/mL. By comparison of the structural characteristics between these compounds, a preliminary structure–activity relationship is discussed. The hydroxy group at C-3′ in **2** would probably increase both anti-osteoclastogenic and antibacterial activity against MRSA, *M. variabilis*, and *M. jannaschii.* Moreover, the chlorine atom at C-4 in **4** would probably promote the activity against MRSA, *M. jannaschii*, and *V. pelagius*.

## 3. Materials and Methods

### 3.1. General Experimental Procedures

UV and IR spectra were recorded on an UV Thermo Fisher scientific Evolution 350 spectrometer (Thermo Fisher Scientific Corporation, Waltham, MA, USA) and an IR Affinity-1 spectrometer, respectively (Shimadzu Corporation, Nakagyo-ku, Kyoto, Japan). ECD spectra were measured on a JASCO J-1500 polarimeter (JASCO Corporation, Tokyo, Japan). The NMR spectra were obtained on a Bruker Avance spectrometer (Bruker BioSpin, Fällanden, Switzerland) operating at 500 or 700 MHz for ^1^H NMR, and 125 or 175 MHz for ^13^C NMR, using TMS as an internal standard. HR-ESIMS spectra were collected on a Waters Xevo G2-S TOF mass spectrometer (Waters Corporation, USA). X-ray diffraction intensity data were performed on an XtalLAB PRO single-crystal diffractometer using Cu K*α* radiation (Rigaku, Japan). TLC and column chromatography (CC) were performed on plates precoated with silica gel GF254 (10–40 μm) and over silica gel (200–300 mesh) (Qingdao Marine Chemical Factory, China), respectively. All solvents employed were of analytical grade (Tianjin Damao Chemical and Industry Factory, Tianjin, China). Semi-preparative high-performance liquid chromatography (Semi-pre HPLC) was performed on a Shimadzu Prominence-I LC 2030 system (Shimadzu, Tokyo, Japan), equipping with an ODS column (YMC-pack ODS-A, YMC Co. Ltd., Kyoto, Japan, 10 × 250 mm, 5 μm, 2.5 mL/min). The artificial sea salt was a commercial product (Guangzhou Haili Aquarium Technology Company, Guangzhou, China).

### 3.2. Fungal Strain and Fermentation

The strain GXIMD 02505 was isolated from a coral, *Pocillopora damicornis,* that was collected from the Weizhou Islands coral reef in Guangxi Zhuang autonomous region, China, in March 2019. It was taxonomically identified as *Aspergillus unguis* GXIMD 02505 by sequence analysis of the internal spacer (ITS) region of the rDNA (GenBank accession no. OL989238). A voucher specimen was deposited in our lab. The strain GXIMD 02505 was cultured on Müller Hinton broth (MB) agar plates (malt extract 15 g, artificial sea salt 15 g, and agar 20 g) at 25 °C for 7 days. Then, it was inoculated in the seed medium (malt extract 15 g and artificial sea salt 15 g in 1.0 L tap distilled H_2_O, pH 7.4–7.8) at 25 °C on a rotary platform shaker at 180 rpm for 48 h. Subsequently, a large-scale fermentation of A. unguis GXIMD 02505 was carried out in modified rice solid medium (150 g rice, 2.7 g malt extract, 2.7 g artificial sea salt, 1.8 g bacteriological peptone, and 180 mL H_2_O) employing 1 L × 72 Erlenmeyer flasks at room temperature for 60 days. The whole fermented cultures were extracted with EtOAc three times to provide a brown extract (94 g).

### 3.3. Extraction and Isolation

The rice fermentation products were extracted with ethyl acetate (EtOAc) and evaporated in vacuo to obtain the crude extract (94 g). The EtOAc crude extract was fractionated by medium pressure liquid chromatography (MPLC) using a step gradient elution with petroleum ether/CH_2_Cl_2_/MeOH (petroleum ether/CH_2_Cl_2_, 1:0–0:1; CH_2_Cl_2_/methanol, 1:0–1:1, *v/v*), which afforded 10 fractions (Frs.1~10) based on TLC (GF_254_) properties. Fr.2 was separated into 14 subfractions (Frs.2-1~2-14) via reversed-phase MPLC with MeOH/H_2_O (10~100%) and then Fr.2-7 was purified by semipreparative high performance liquid chromatography (HPLC) with MeOH/H_2_O (83:17, *v/v*, 5.0 mL/min) to yield compound **8** (*t*_R_ = 42 min, 1.5 mg). Fr.2-9 was further purified by semipreparative HPLC on a YMC ODS column eluting with MeOH/H_2_O (83:17, *v/v*, adding 0.02% trifluoroacetic acid (TFA), 2.0 mL/min) to obtain compound **4** (*t*_R_ = 37 min, 17 mg). Fr.2-13 was purified by semipreparative HPLC on naphthyl column with MeOH/H_2_O (81:19, *v/v*, adding 0.02%TFA, 2.0 mL/min) to yield compounds **3** (*t*_R_ = 54 min, 1.6 mg) and **5** (*t*_R_ = 58 min, 8.4 mg). Besides, Fr.3 was directly separated by semipreparative HPLC with MeOH/H_2_O (87:13, *v/v*, 5.0 mL/min) to obtained compounds **1** (*t*_R_ = 36 min, 17 mg), **2** (*t*_R_ = 20 min, 22 mg), **6** (*t*_R_ = 31 min, 12 mg), and **7** (*t*_R_ = 57 min, 11 mg).

Aspergillusidone H (**3**). white amorphous solid; UV (MeOH) λ_max_ (logε) 270 (2.82), 207 (3.43) nm; IR (film) *ν*_max_ 3446, 1683, 1653, 1269, 1205, 1139 cm^−1^; ^1^H and ^13^C NMR data, Table 1; HR-ESIMS *m/z* 389.1159 [M + H]^+^ (calcd for C_21_H_22_ClO_5_, 389.1156), 411.0967 [M + Na]^+^ (calcd for C_21_H_21_ClNaO_5_, 411.0975).

### 3.4. Computational Methods

The calculated ECD curve of **8** was performed by the Gaussian 16 software, referred to in our previously reported method [18]. In brief, Merck molecular force field (MMFF) calculations were carried out by means of the Spartan´14 software (Wavefunction Inc., Irvine, CA, USA). Low-energy conformers with a Boltzmann distribution over 1% were chosen for DFT/TD-DFT calculations at the B3LYP/6-311+G (d, p)//B3LYP/6-31+G (d) level in methanol by adopting 50 excited states. The ECD data were generated by the SpecDis 3.0 (University of Wurzburg, Wurzburg, Germany) using a half band width of 0.19 eV and shifted by –11 nm to facilitate comparison to the experimental data.

### 3.5. X-ray Crystallography

The crystallographic data of compound **2** obtained in MeOH was collected on a Rigaku XtaLAB PRO single-crystal diffractometer using Cu K*α* radiation (λ = 1.54178 Å). Briefly, its X-ray crystal structure was solved by direct methods using SHELXS97, expanded by difference Fourier techniques, and refined by full-matrix least-squares calculation finally. The non-hydrogen atoms were refined anisotropically, and all hydrogen atoms were fixed at the geometrically ideal positions.

Crystal data for aspergillusether F (**2**): C_18_H_17_Cl_3_O_4_•4CH_3_OH, *M*r = 499.79, crystal size 0.15 × 0.05 × 0.06 mm^3^, triclinic, *a* = 10.2737(4) Å, *b* = 10.5413(10) Å, *c* = 12.6374(8) Å, *α* = 109.081(7)°, *β* = 111.110(5)°, *γ* = 90.805(5)°, *V* = 1193.05(16) Å^3^, *T* = 100.00(10) K, space group *P*-1, *Z* = 2, *μ*(CuK*α*) = 1.391 mm^−1^, 10939 reflections collected, 4203 independent reflections (*R*_int_ = 0.0643). The final *R*_1_ values were 0.0751 (*I* > 2*σ*(*I*)). The final w*R*(*F*^2^) values were 0.2040 (*I* > 2*σ*(*I*)). The final *R*_1_ values were 0.0880 (all data). The final w*R*(*F*^2^) values were 0.2175 (all data). The goodness of fit on *F*^2^ was 0.999. The crystallographic data for the structure of aspergillusether F have been deposited in the Cambridge Crystallographic Data Centre (deposition number: CCDC 2130596).

### 3.6. Anti-Osteoclastogenic Assay

These isolates were evaluated for their inhibitory activities of LPS-induced NF-κB activation in RAW264.7 cells, as detected by a luciferase reporter gene assay as described previously [4]. In brief, the RAW264.7 cells, stably transfected with NF-κB luciferase reporter gene, were plated in triplicate for all treatments and controls in 96-well plates, and then pretreated with these compounds (20 μM) and BAY11-7082 (NF-κB inhibitor as positive control, 5 μM, Sigma-Aldrich) for 30 min, followed by 5 μg/mL LPS stimulation for 8 h. Cells were harvested, and luciferase activities were measured by the luciferase assay system (Promega, Madison, WI, USA). The dose-dependent effects of compounds (1, 5, 10, 20, and 50 μM) on LPS induced NF-κB luciferase activity were also assayed by the same procedure.

For further exploration of the potential inhibition on osteoclastogenesis by compounds **2** and **7**, concentrations of 5 and 15 μM were added in BMMs with both murine macrophage-stimulating factor (M-CSF) (50 ng/mL) and RANKL (100 ng/mL) stimulation for 3 days. Then, the cells were fixed and stained for tartrate-resistant acidic phosphatase activity (TRAP) and images were taken by an inverted microscope (Nikon, Japan). A CCK-8 kit was used to evaluate the cytotoxic effects of **2** and **7** on BMMs. BMMs (1 × 10^5^ cells/mL) with M-CSF (50 ng/mL) were seeded with **2** and **7** (0, 1, 5, 15, 20 μM) in 96-well plate for 72 h. Besides, NF-κB p65 nuclear translocation assay by compound **7** was performed by a confocal microscope as described previously [4]. Data are expressed as the mean ± SD and analyzed using GraphPad Prism 7.0 software (San Diego, CA, USA). Statistical differences among groups were performed using one-way analysis of variance (ANOVA) with the Bonferroni *post-hoc* test. A *p*-value of <0.05 was considered statistically significant.

### 3.7. Antibacterial Assay

Antibacterial effects against a panel of marine biofilm-forming bacterial strains, including methicillin-resistant *Staphylococcus aureus*, *Pseudomonas aeruginosa*, *Vibrio parahemolyticus*, *V. alginolyticus*, *V. tubiashii*, *Microbulbifer variabilis*, *Marinobacterium jannaschii*, *V. pelagius*, *V. rotiferianus*, and *Alteromonas macleodii*, were tested using our previously reported method [30]. Chloramphenicol, nalidixic acid, streptomycin, and/or ampicillin were used as positive controls.

### 3.8. Molecular Docking

The Schrödinger 2019-4 suite (Schrödinger Inc., New York, NY, USA) was employed to perform the docking study [18]. The crystal structure of human NF-κB p65 was obtained from Protein Data Bank (http://www.pdb.org, accessed on 10 January 2022) (PDB code: 3GUT, chain A). The initial structure of protein was first automatically corrected by “Protein Preparation” module. Then, the binding site was putatively similar to the pocket of HIV-1 LTR, which was included in the crystal structure. The ligands were then flexibly docked to the pocket by the Glide module with standard precision mode. The docking pose with best glide score was chosen for presenting the bind mode of the molecule. The PyMOL software (DeLano Scientific, Palo Alto, CA, USA) was used to obtain the 3D structures of the binding models.

## 4. Conclusions

The chemical investigation of the Beibu Gulf coral-derived fungus *Aspergillus unguis* GXIMD 02505 led to the characterization of eight chlorinated aromatic polyketides, including a new depsidone derivative (**3**), together with seven known biosynthetically related analogs. The X-ray crystal structure of **2** and the absolute configuration of **8** assigned by ECD calculations are described herein for the first time. Compounds **1**−**5**, **7** and **8** displayed inhibition of LPS-induced NF-κB in RAW 264.7 macrophages at 20 μM. Moreover, the two potent inhibitors (**2** and **7**) further dose-dependently suppressed RANKL-induced osteoclast differentiation without any evidence of cytotoxicity in BMMs. Compound **7** could block RANKL-induced NF-κB p65 nuclear translocation. Besides, compounds **1**, **2**, **4**, and **6**−**8** showed inhibitory activity against MRSA, *M. variabilis*, *M. jannaschii*, and *V. pelagius*, with their MIC values ranging from 2 to 64 μg/mL. The structure–activity relationship is primarily discussed. This is the first report to report the inhibitory activity of these chlorinated aromatic polyketides on osteoclast differentiation. Our findings would not only expand the structural diversity of chlorinated depsidones, but also provide a basis for discovering lead compounds to treat skeletal diseases characterized by excessive osteoclast differentiation, as well as for use as anti-fouling agents.

## Figures and Tables

**Table 1:** ^1^H (700 MHz) and ^13^C (175 MHz) NMR Data for **3** (CD_3_OD).

Position	*δ*_C_, Type	*δ*_H_ (*J* in Hz)	HMBC
1	144.0, C		
1-Me	21.6, CH_3_	2.45, s	1, 2, 11a
2	111.2, CH	6.73, s	3, 4, 11a
3	163.0, C		
3-OMe	56.4, CH_3_	3.87, s	3
4	116.1, C		
4-Me	10.2, CH_3_	2.15, s	3, 4, 4a
4a	162.3, C		
5a	143.6, C		
6	135.7, C		
7	117.3, C		
8	143.7, C		
9	117.8, C		
9-Me	9.2, CH_3_	2.24, s	8, 9a, 9
9a	150.2, C		
11	165.3, C		
11a	114.2, C		
1′	131.8, C		
2′	128.5, CH	5.40, q (6.8)	6, 3′, 4′
3′	14.0, CH_3_	1.82, d (6.8)	1′
4′	17.7, CH_3_	1.91, s	6, 1′

**Table 2:** Antibacterial activities of compounds **1**–**8**.

Compound	Minimum Inhibitory Concentration (MIC, μg/mL)			
	MRSA	*M. variabilis*	*M. jannaschii*	*Pelagius*
1	16	32	64	-
2	2	16	32	-
3	-	-	-	-
4	2	8	16	64
5	>128	128	-	-
6	32	8	32	-
7	16	64	>128	-
8	>128	8	32	-
control	1 ^d^	1 ^a^ 16 ^b^ 32 ^c^	1 ^a^ 16 ^b^ 8 ^c^	1 ^b^

“-” = Inactive, ^a^ Chloramphenicol, ^b^ Nalidixic acid, ^c^ Streptomycin, ^d^ Ampicillin.

## Supplementary Materials

The following supporting information can be downloaded at: https://www.mdpi.com/article/10.3390/md20030178/s1. The NMR, HR-ESIMS, UV, and IR spectra of compounds **1** and **3** (Figures S1−S18), the X ray crystal structure of compound **2** (Figure S19), the calculated ECD data of **8** (Figure S20 and Tables S1 and S2), physicochemical data of known compounds **2**, **4**–**8**, antibacterial activity by agar diffusion method (Figures S21−S28) and the ITS sequence of *A. unguis* GXIMD 02505.
