# Supplementary material for: Anti-Osteoclastogenic and Antibacterial Effects of Chlorinated Polyketides from the Beibu Gulf Coral-Derived Fungus Aspergillus unguis GXIMD 02505
Source: Mar Drugs. 2022 Feb 28;20(3):178. doi: 10.3390/md20030178 (PMC8956104; doi:10.3390/md20030178)
Supplement: Supplementary file 1 [file marinedrugs-20-00178-s001.zip › marinedrugs-1577167-New-SI.pdf]

Supplementary Materials

Anti-osteoclastogenic and Antibacterial Effects of Chlorinated Polyketides from the Beibu Gulf Coral-derived Fungus *Aspergillus unguis* GXIMD 02505

Yanting Zhang [1,†], Zhichao Li[2,†], Bingyao Huang [1], Kai Liu [1], Shuai Peng [1], Xinming Liu [1], Chenghai Gao [1], Yonghong Liu [1,*], Yanhui Tan [2,*], and Xiaowei Luo [1,*]

[1] Institute of Marine Drugs, Guangxi University of Chinese Medicine, Nanning 530200, China;

[2] State Key Laboratory for Chemistry and Molecular Engineering of Medicinal Resources, School of Chemistry and Pharmaceutical Sciences, Guangxi Normal University, Guilin 541004, China;

* Correspondence: yonghongliu@scsio.ac.cn (Y.L.); tyh533@126.com (Y.T.); luoxiaowei1991@126.com (X.L.).

† These authors contributed equally to this work.

Contents of Supporting Information

Figure S1. ^{1}H NMR spectrum of Aspergillusether J (**1**) (methanol-d_4, 500 MHz)....3
Figure S2. ^{13}C NMR spectrum of Aspergillusether J (**1**) (methanol-d_4, 125 MHz)....4
Figure S3. DEPT 135 NMR spectrum of Aspergillusether J (**1**) (methanol-d_4, 125 MHz).....5
Figure S4. HSQC spectrum of Aspergillusether J (**1**) (methanol-d_4)....6
Figure S5. HMBC spectrum of Aspergillusether J (**1**) (methanol-d_4)....7
Figure S6. NOESY spectrum of Aspergillusether J (**1**) (methanol-d_4)....8
Figure S7. HRESIMS spectrum of Aspergillusether J (**1**)....9
Figure S8. UV spectrum of Aspergillusether J (**1**)....10
Figure S9. IR spectrum of Aspergillusether J (**1**)....10
Figure S10. ^{1}H NMR spectrum of Aspergillusidone H (**3**) (methanol-d_4, 700 MHz)....11
Figure S11. ^{13}C NMR spectrum of Aspergillusidone H (**3**) (methanol-d_4, 175 MHz)....12
Figure S12. DEPT 135 NMR spectrum of Aspergillusidone H (**3**) (methanol-d_4, 175 MHz)....13
Figure S13. HSQC spectrum of Aspergillusidone H (**3**) (methanol-d_4)....14
Figure S14. HMBC spectrum of Aspergillusidone H (**3**) (methanol-d_4)....15
Figure S15. NOESY spectrum of Aspergillusidone H (**3**) (methanol-d_4)....16
Figure S16. HRESIMS spectrum of Aspergillusidone H (**3**)....17
Figure S17. UV spectrum of Aspergillusidone H (**3**)....18
Figure S18. IR spectrum of Aspergillusidone H (**3**)....18
Figure S19. X ray crystal structure of compound **2**.....19
Table S1. Energies of **8** at MMFF94 force field....19
Table S2. Energies of **8** at B3LYP/6–31+g(d) level in methanol.....20
Figure S20. The optimized conformers and equilibrium populations of **8**.....23
Physicochemical data of known compounds **2**, **4–8.**....23
Figure S21. Anti-MRSA activity of compounds **1–8** by agar diffusion method. (CHL: Chloramphenicol; SM: Streptomycin; AMP: Ampicillin)....25
Figure S22. Anti-*M. variabilis* activity of compounds **1–8** by agar diffusion method.....25
Figure S23. Anti-*M. jannaschii* activity of compounds **1–8** by agar diffusion method.....26
Figure S24. Anti-*V. Pelagius* activity of compound **4** by agar diffusion method.....26
Figure S25. Anti-MRSA activity of compounds **1**, **2**, **4**, **6**, **7** and ampicillin.....26
Figure S26. Anti-*M. variabilis* activity of compounds **1**, **2**, **4**, **6~8** and positive control....27
Figure S27. Anti-*M. jannaschii* activity of compounds **1**, **2**, **4**, **6**, **8** and positive control....27
Figure S28. Anti-*V. Pelagius* activity of compounds **4** and nalidixic acid....28
The ITS sequence of *Aspergillus unguis* GXIMD 02505....28

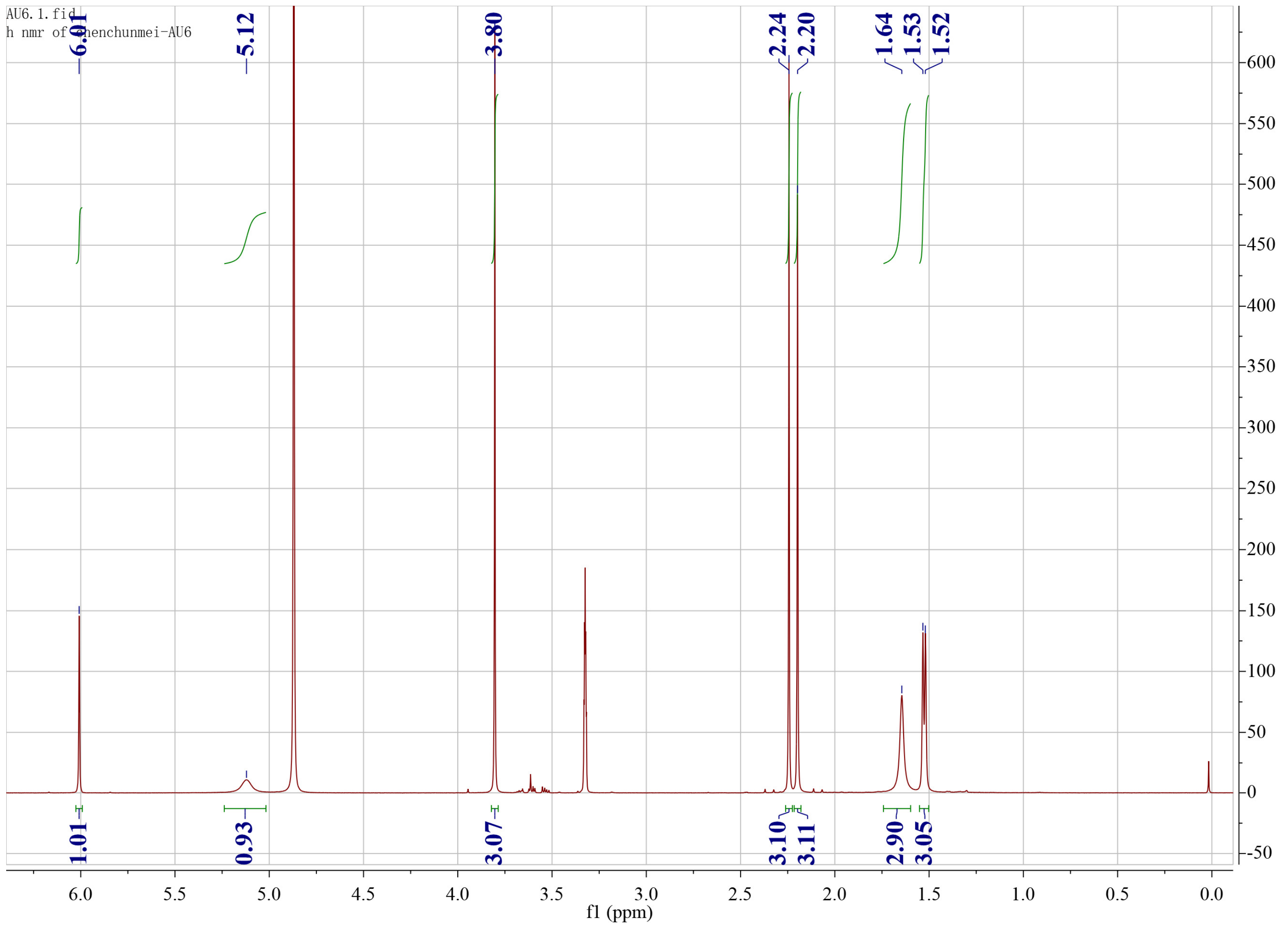

Figure S1. ^{1}H NMR spectrum of Aspergillusether J (**1**) (methanol-d_4, 500 MHz)

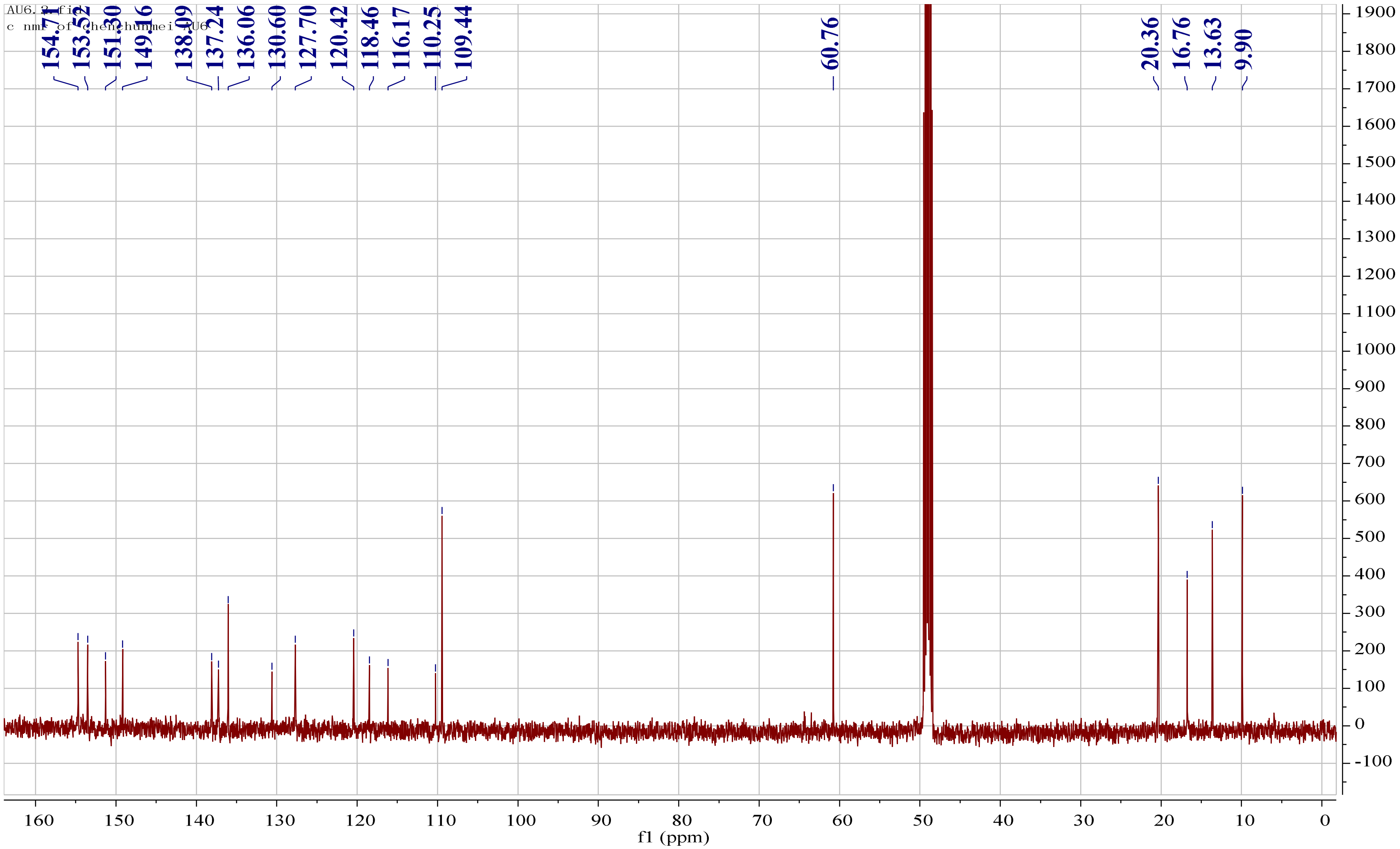

Figure S2. ^{13}C NMR spectrum of Aspergillusether J (**1**) (methanol-d_4, 125 MHz)

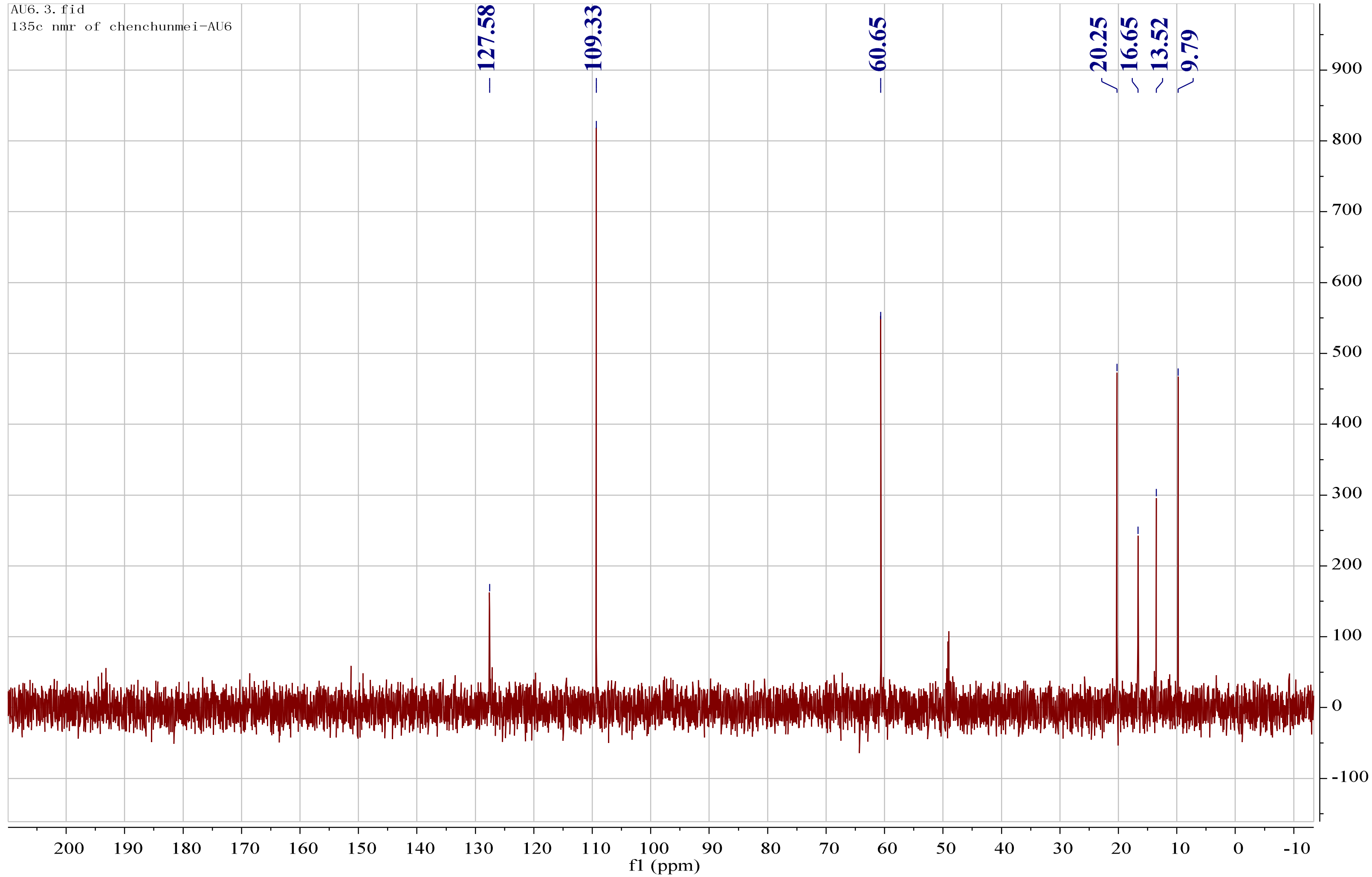

Figure S3. DEPT 135 NMR spectrum of Aspergillusether J (**1**) (methanol-d_4, 125 MHz)

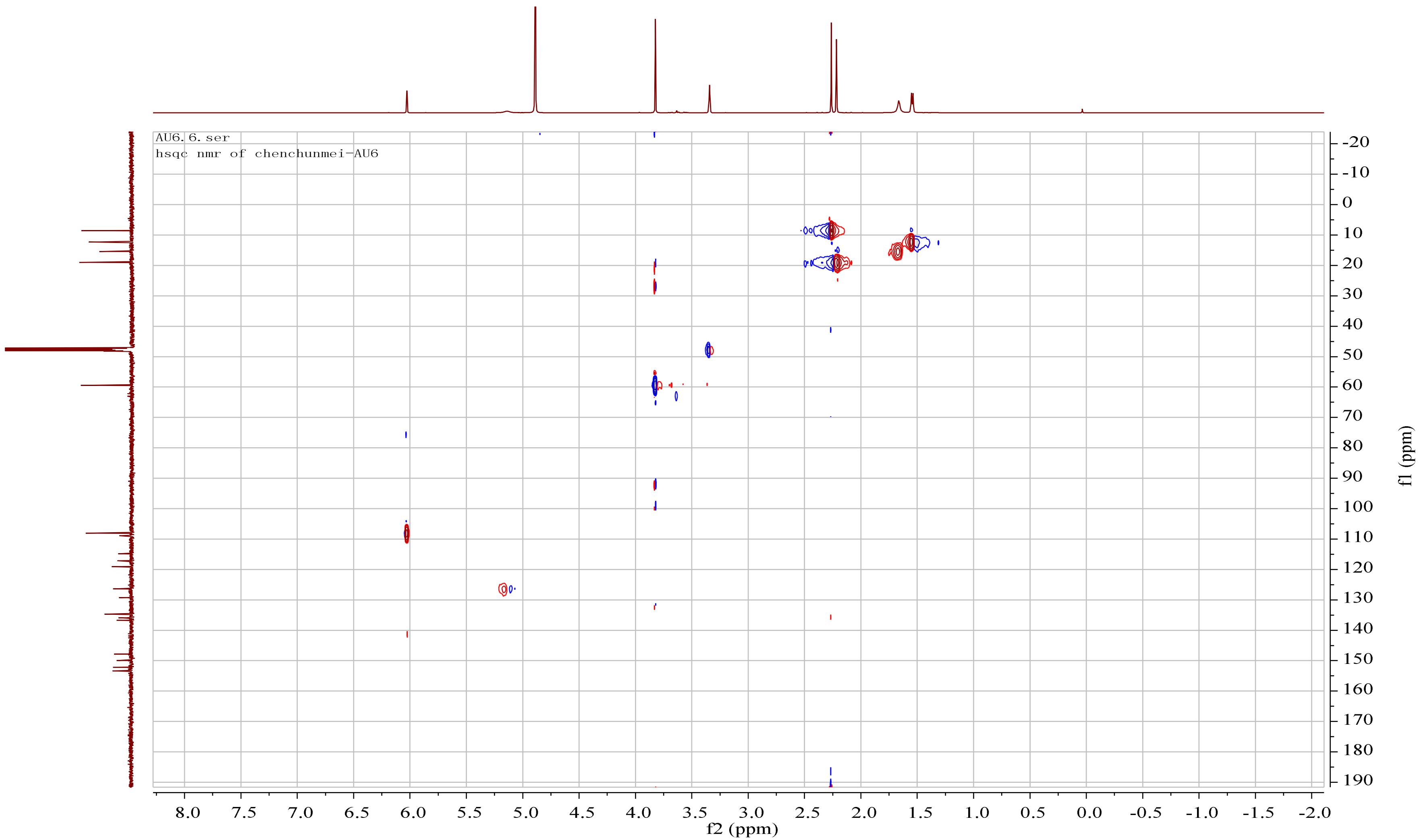

Figure S4. HSQC spectrum of Aspergillusether J (**1**) (methanol-d_4)

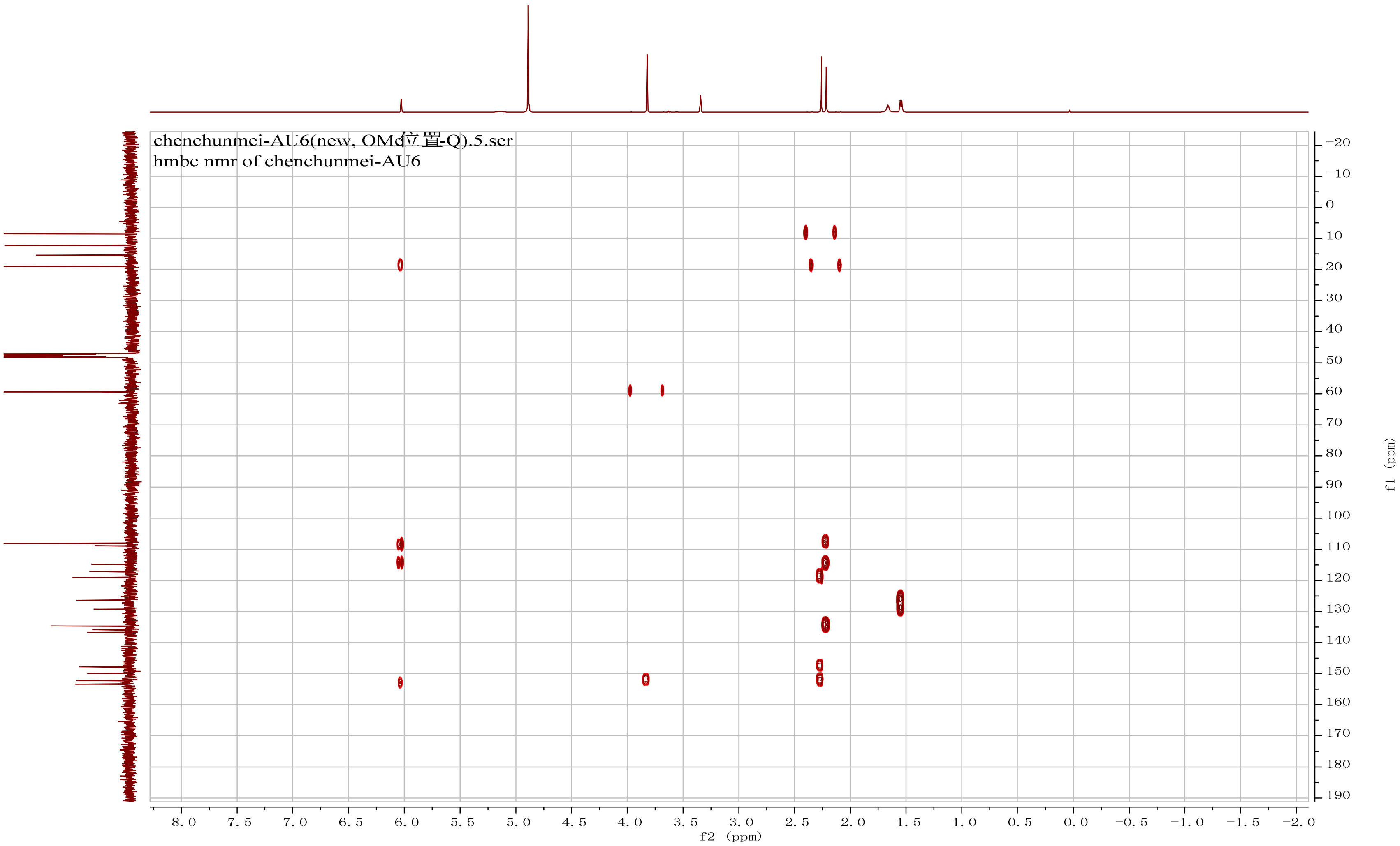

Figure S5. HMBC spectrum of Aspergillusether J (**1**) (methanol-d_4)

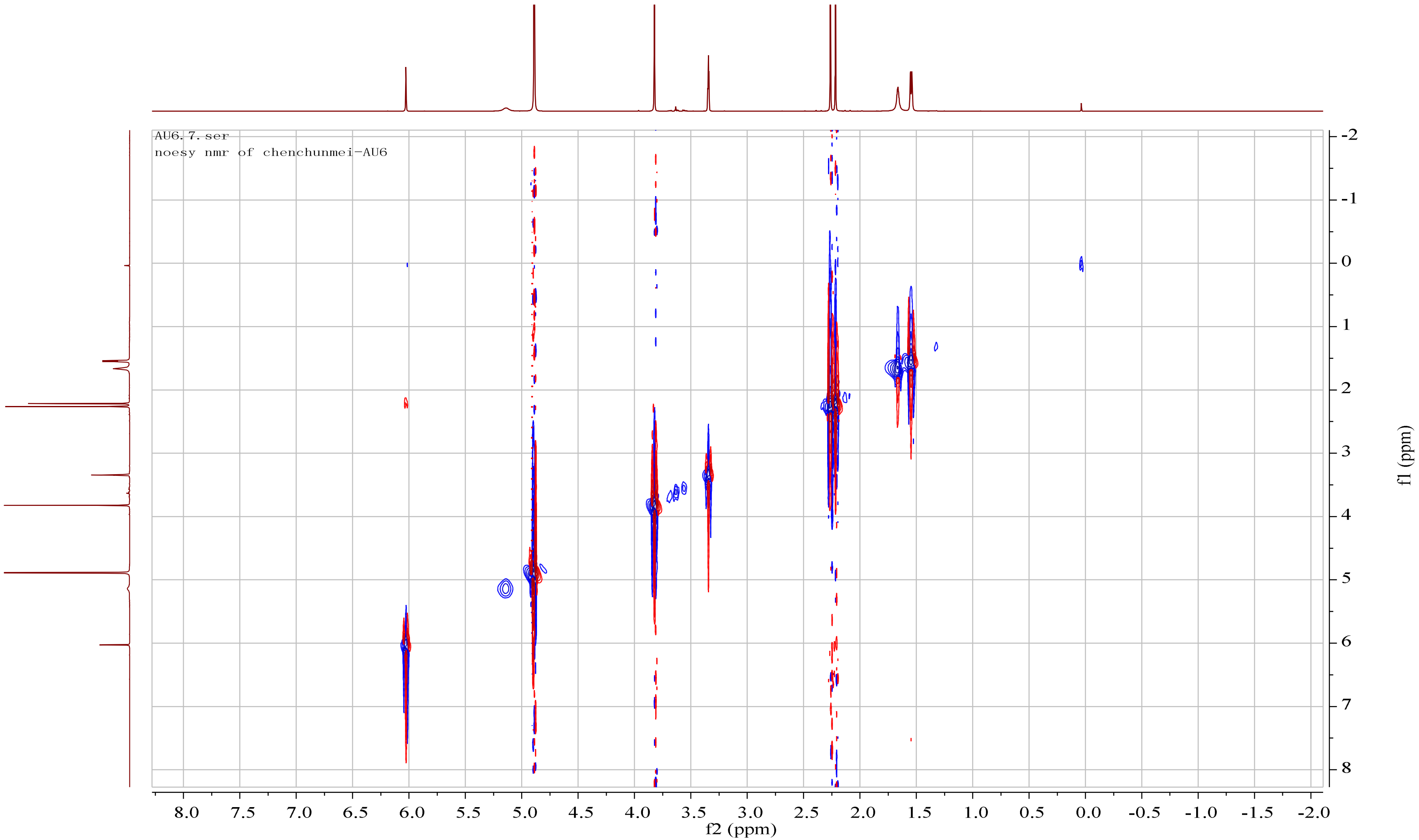

Figure S6. NOESY spectrum of Aspergillusether J (**1**) (methanol-d_4).

Mass Spectrum SmartFormula Report

Analysis Info

Analysis Name	D:\Data\MS\data\202105\chenchunmei_AU6_neg_11_01_10842.d	Acquisition Date	5/28/2021 5:29:30 PM
Method	LC_Direct Infusion_neg_70-500mz.m	Operator	SCSIO
Sample Name	chenchunmei_AU 6_neg	Instrument	maXis 255552.00029
Comment			

Acquisition Parameter

Source Type	ESI	Ion Polarity	Negative	Set Nebulizer	0.4 Bar
Focus	Active	Set Capillary	4000 V	Set Dry Heater	180 °C
Scan Begin	100 m/z	Set End Plate Offset	-500 V	Set Dry Gas	4.0 l/min
Scan End	1500 m/z	Set Charging Voltage	0 V	Set Divert Valve	Waste
		Set Corona	0 nA	Set APCI Heater	0 °C

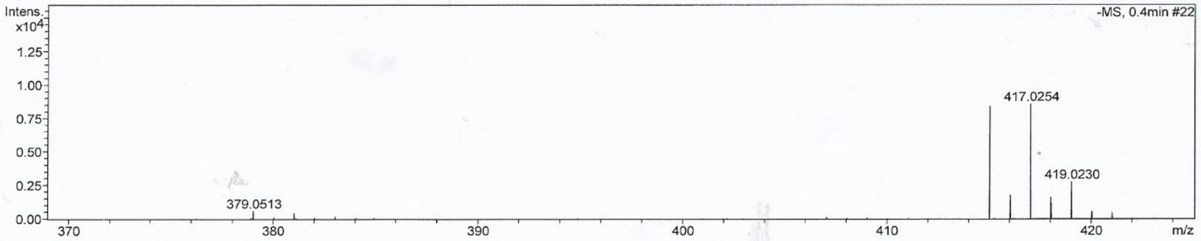

Meas. m/z	#	Ion Formula	Score	m/z	err [ppm]	err [mDa]	mSigma	rdb	e⁻ Conf	N-Rule
379.0513	1	C19H17Cl2O4	100.00	379.0509	0.9	0.4	40.4	10.5	even	ok
415.0285	1	C19H18Cl3O4	100.00	415.0276	-2.0	-0.8	12.2	9.5	even	ok

chenchunmei_AU 6_neg_11_01_10842.d
Bruker Compass DataAnalysis 4.1 printed: 5/28/2021 5:33:45 PM by: SCSIO Page 1 of 1

Figure S7. HRESIMS spectrum of Aspergillusether J (**1**).

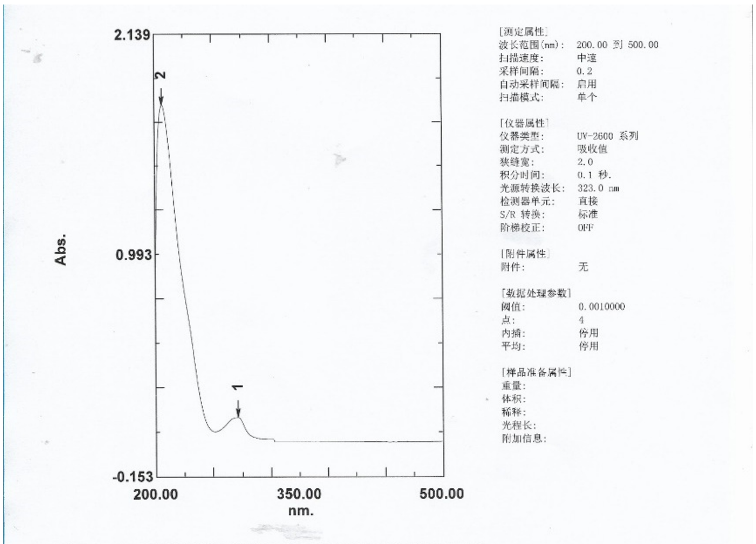

No.	P/V	波长(nm)	Abs.	描述
1	⬆	285.40	0.151	
2	⬆	207.[illegible]	1.770	

Figure S8. UV spectrum of Aspergillusether J (**1**).

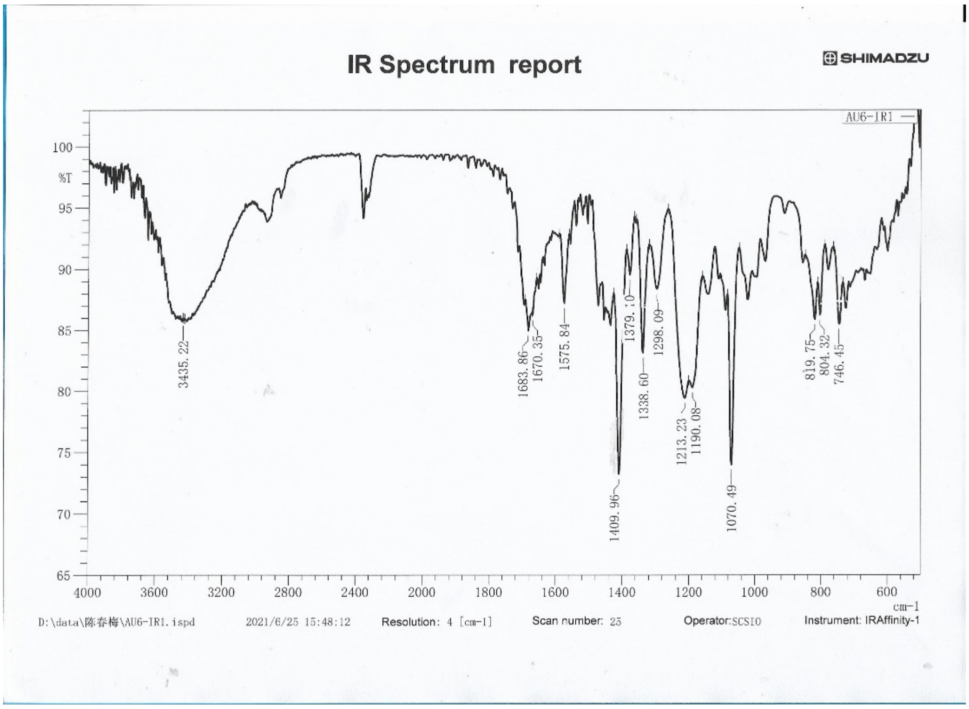

Figure S9. IR spectrum of Aspergillusether J (**1**)

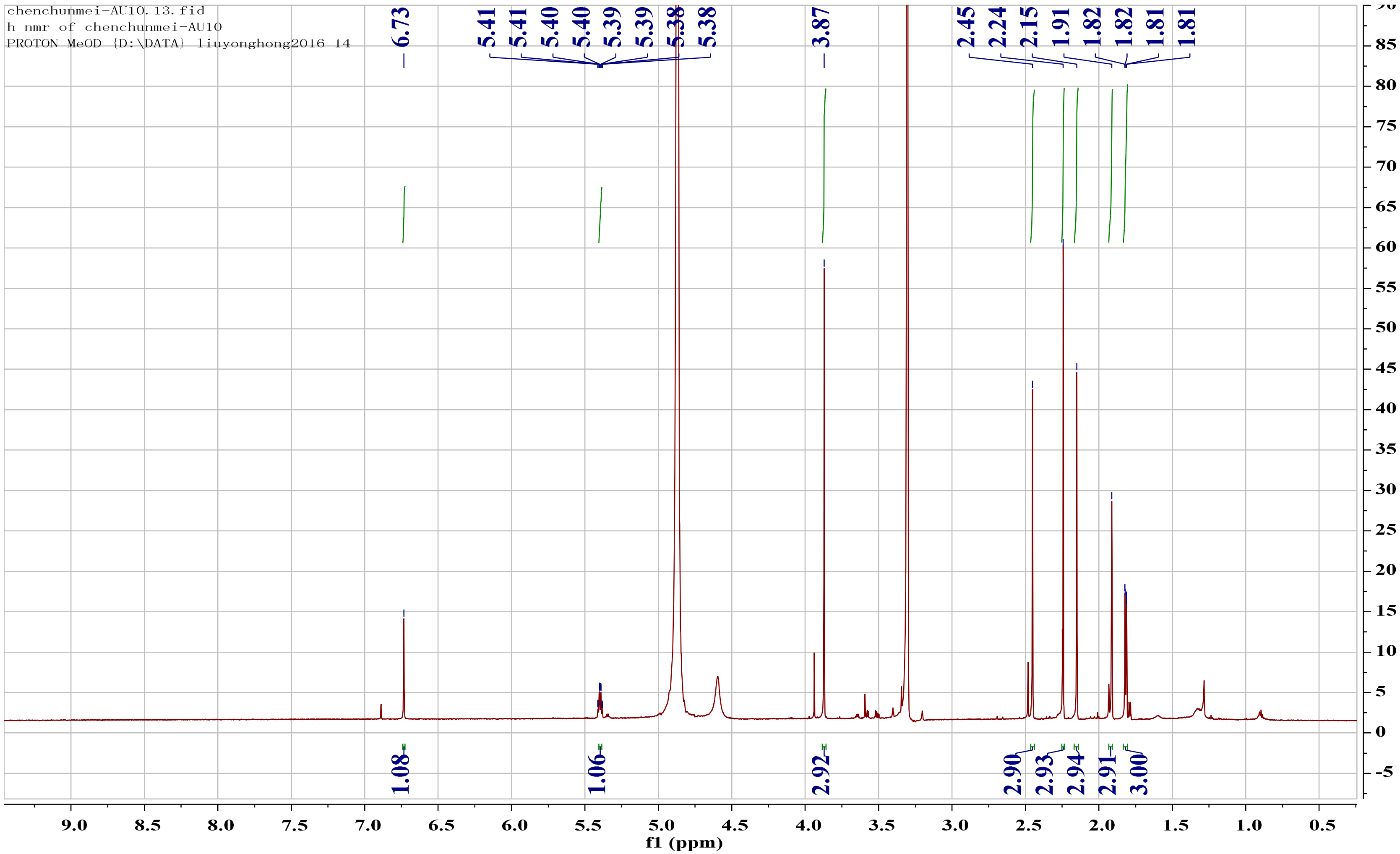

Figure S10. ^{1}H NMR spectrum of Aspergillusidone H (**3**) (methanol-d_4, 700 MHz)

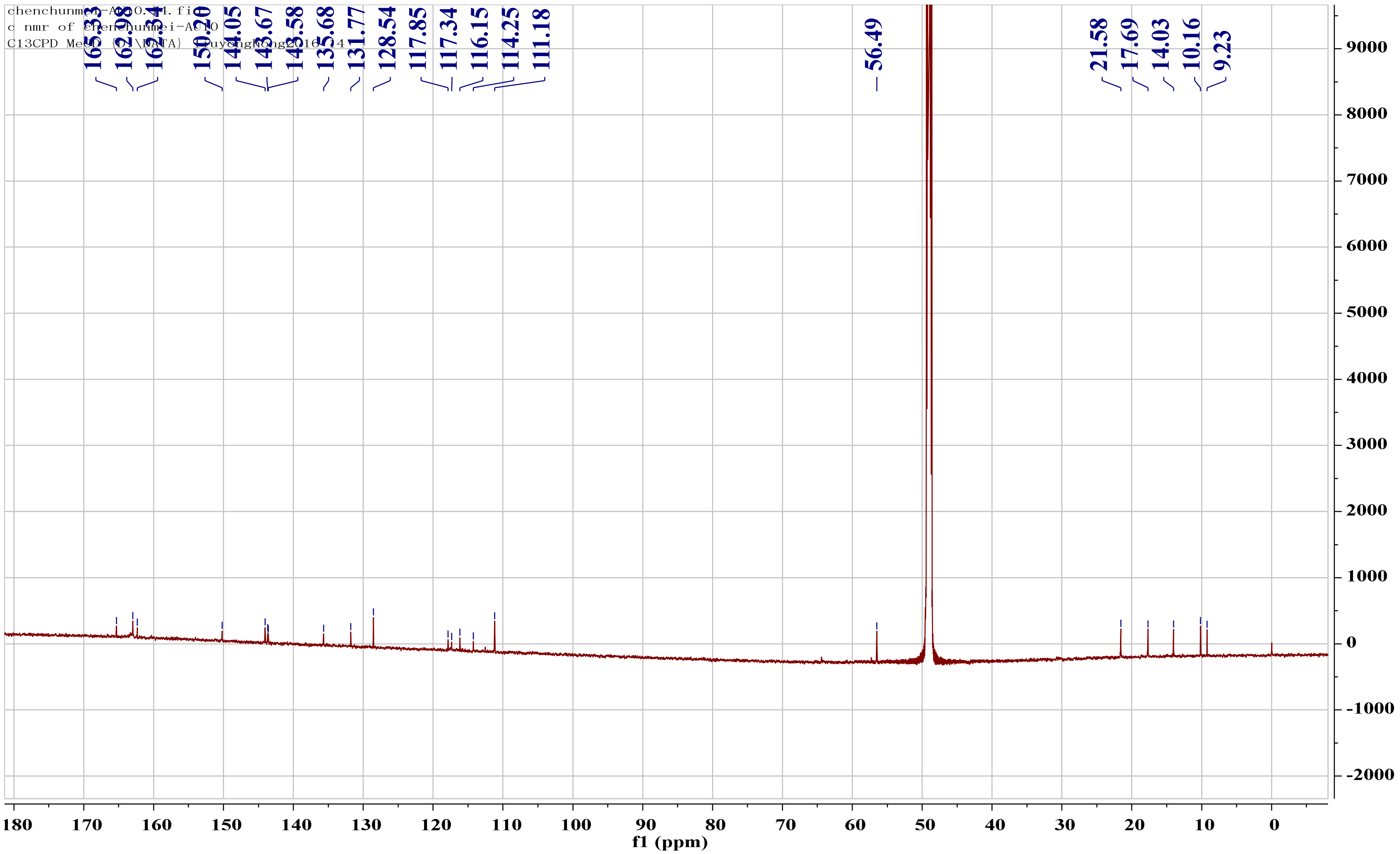

Figure S11. ^{13}C NMR spectrum of Aspergillusidone H (**3**) (methanol-*d*$_4$, 175 MHz)

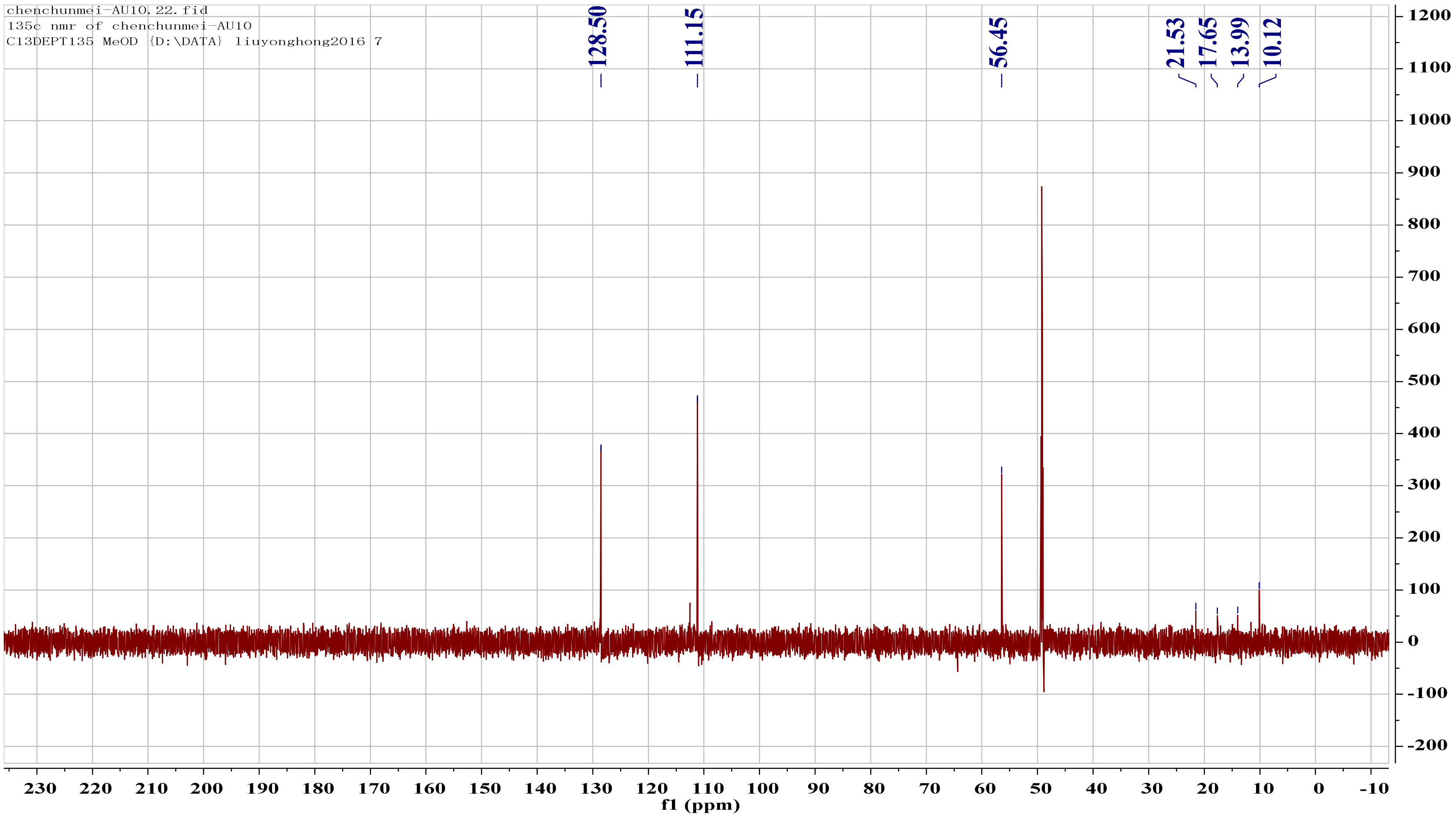

Figure S12. DEPT 135 NMR spectrum of Aspergillusidone H (**3**) (methanol-d_4, 175 MHz)

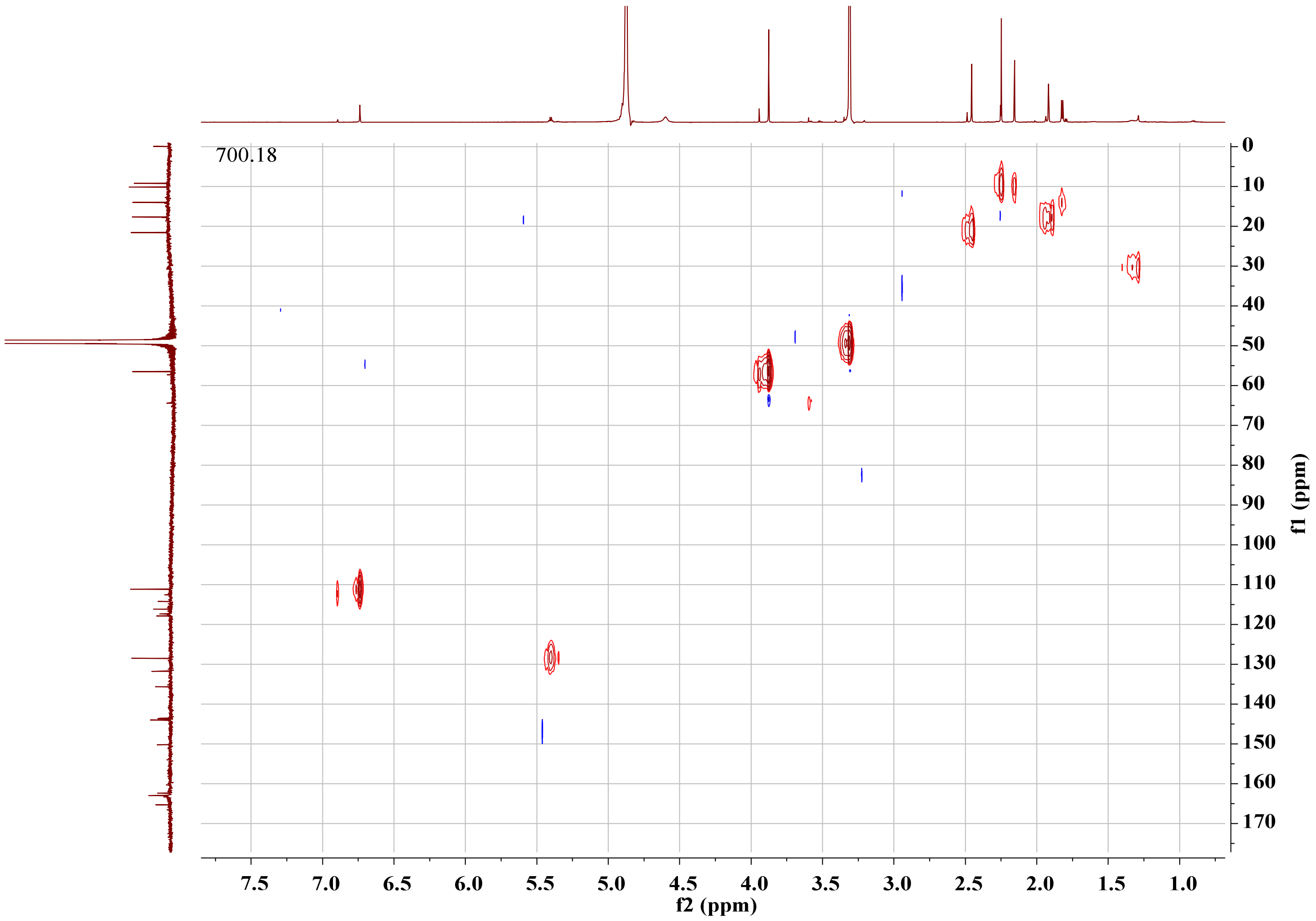

Figure S13. HSQC spectrum of Aspergillusidone H (**3**) (methanol-d_4)

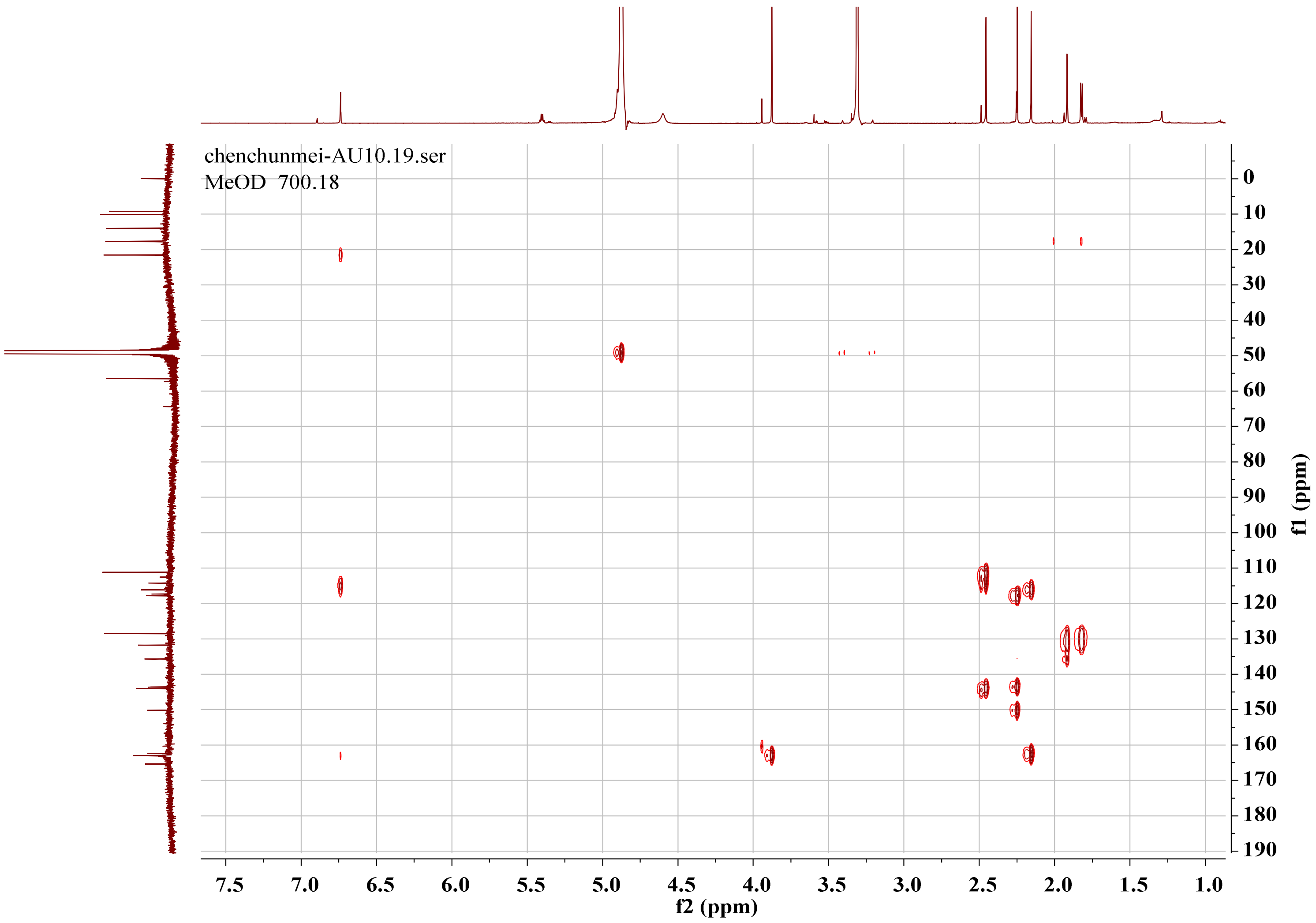

Figure S14. HMBC spectrum of Aspergillusidone H (**3**) (methanol-d_4)

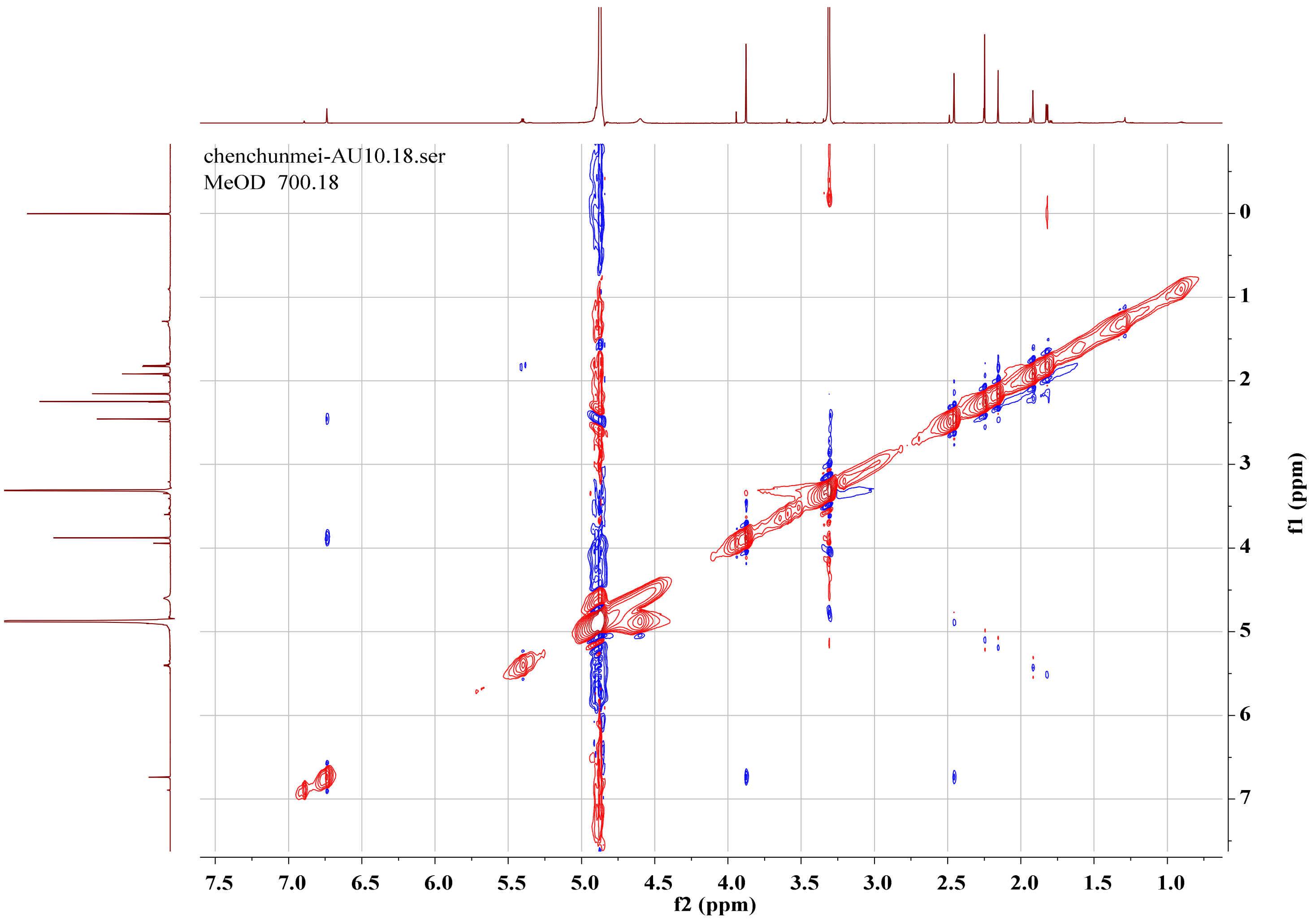

Figure S15. NOESY spectrum of Aspergillusidone H (**3**) (methanol-d_4)

Mass Spectrum SmartFormula Report

Analysis Info

Analysis Name	D:\Data\MS\data\202106\chenchunmei_AU10_pos_22_01_11002.d	Acquisition Date	6/24/2021 8:40:37 AM
Method	LC_Direct Infusion_pos_70-500mz.m	Operator	SCSIO
Sample Name	chenchunmei_AU 10_pos	Instrument	maXis 255552.00029
Comment			

Acquisition Parameter

Source Type	ESI	Ion Polarity	Positive	Set Nebulizer	0.4 Bar
Focus	Active	Set Capillary	4500 V	Set Dry Heater	180 °C
Scan Begin	70 m/z	Set End Plate Offset	-500 V	Set Dry Gas	4.0 l/min
Scan End	1500 m/z	Set Charging Voltage	0 V	Set Divert Valve	Waste
		Set Corona	0 nA	Set APCI Heater	0 °C

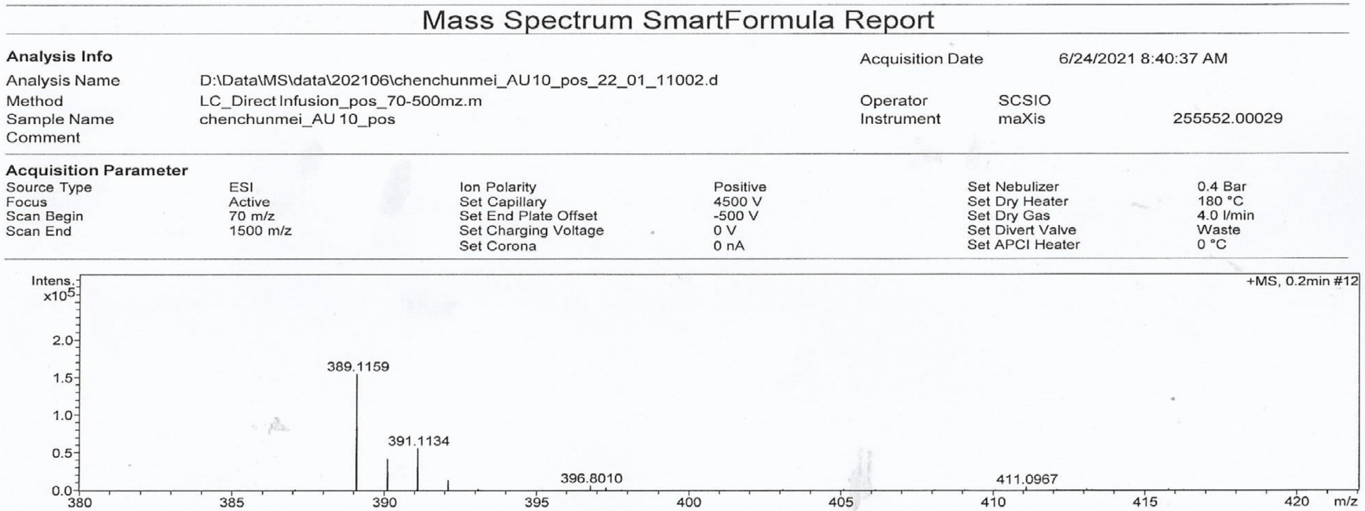

Meas. m/z	#	Ion Formula	Score	m/z	err [ppm]	err [mDa]	mSigma	rdb	e⁻ Conf	N-Rule
389.1159	1	C21H22ClO5	100.00	389.1150	2.2	0.8	18.3	10.5	even	ok
411.0967	1	C21H21ClNaO5	100.00	411.0970	-0.7	-0.3	25.2	10.5	even	ok
777.2248	1	C42H43Cl2O10	100.00	777.2228	2.7	2.1	35.4	20.5	even	ok
799.2043	1	C42H42Cl2NaO10	100.00	799.2047	-0.5	-0.4	45.0	20.5	even	ok

chenchunmei_AU 10_pos_22_01_11002.d
Bruker Compass DataAnalysis 4.1 printed: 6/24/2021 8:44:36 AM by: SCSIO Page 1 of 1

Figure S16. HRESIMS spectrum of Aspergillusidone H (**3**)

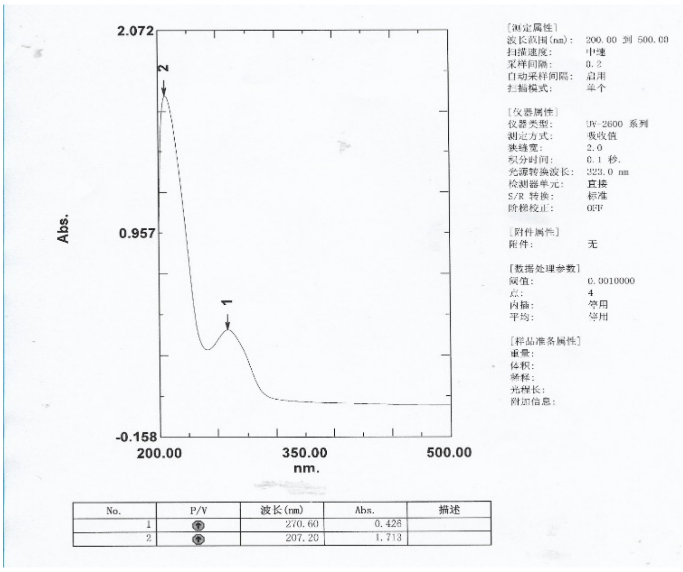

No.	P/V	波长(nm)	Abs.	描述
1	↑	270.60	0.426	
2	↑	207.20	1.713	

Figure S17. UV spectrum of Aspergillusidone H (**3**)

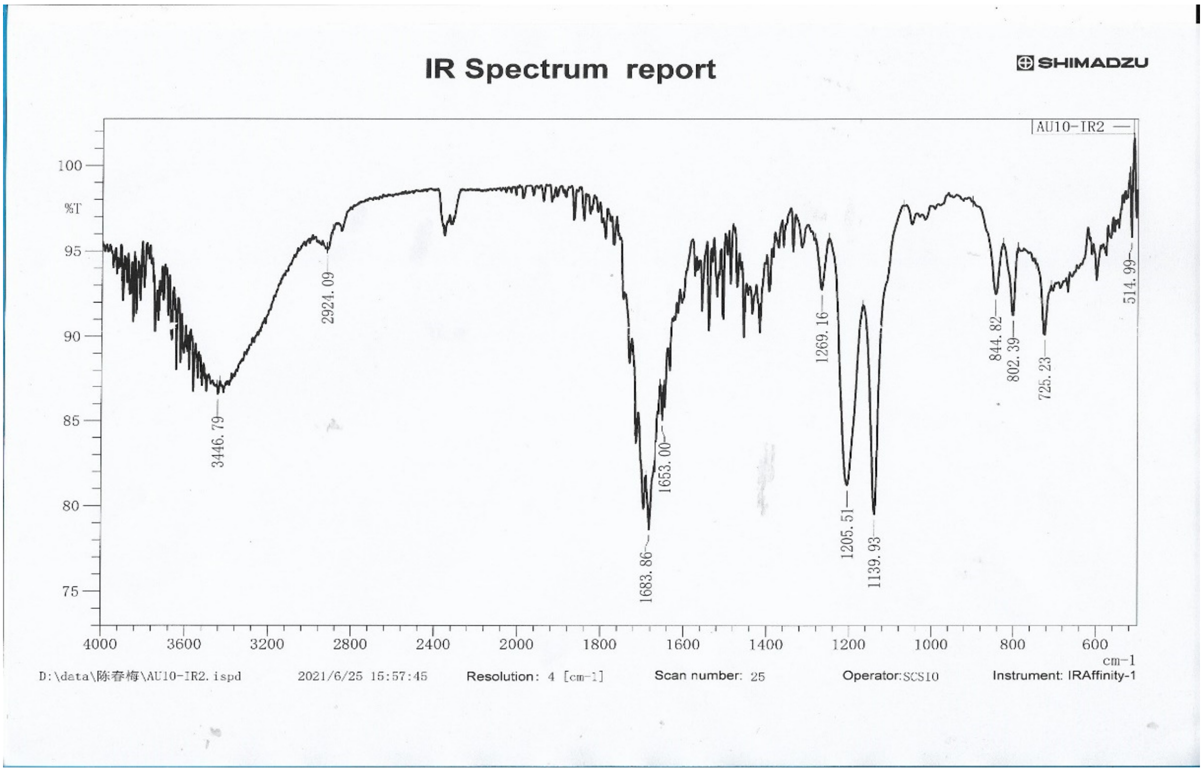

Figure S18. IR spectrum of Aspergillusidone H (**3**)

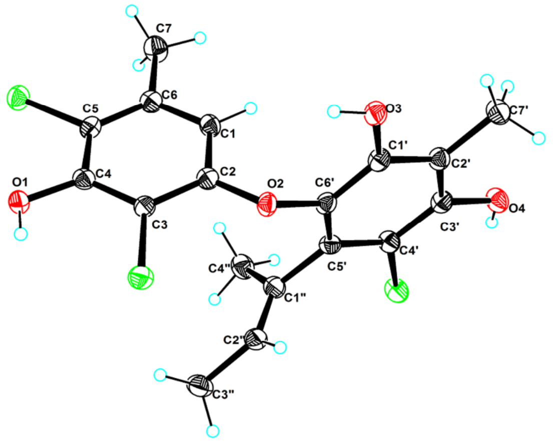

Figure S19. X ray crystal structure of compound **2**.

Table S1. Energies of **8** at MMFF94 force field.

Configuration	Conformer	Energy (kcal/mol)	Population (%)
R-**8**	1	211.00	35.2
R-**8**	2	211.55	28.1
R-**8**	3	215.72	5.2
R-**8**	4	215.73	2.6
R-**8**	5	216.19	4.3
R-**8**	6	216.30	4.1
R-**8**	7	216.54	3.8
R-**8**	8	216.74	3.5
R-**8**	9	217.09	1.5
R-**8**	10	217.86	2.2
R-**8**	11	218.42	0.9
R-**8**	12	218.83	1.5
R-**8**	13	218.94	1.4
R-**8**	14	218.96	0.7
R-**8**	15	219.38	1.2
R-**8**	16	219.44	0.6
R-**8**	17	219.56	1.1
S-**8**	1	211.00	17.0
S-**8**	2	211.55	27.1
S-**8**	3	213.72	11.3
S-**8**	4	214.33	8.9
S-**8**	5	215.16	6.3
S-**8**	6	215.73	5.0
S-**8**	7	216.19	4.2
S-**8**	8	216.30	2
S-**8**	9	216.54	3.6

S-**8**	10	216.74	1.7
S-**8**	11	217.09	2.9
S-**8**	12	217.86	2.1
S-**8**	13	218.42	1.7
S-**8**	14	218.96	1.4
S-**8**	15	219.44	1.1
S-**8**	16	219.56	0.5
S-**8**	17	219.57	1.1

Table S2. Energies of **8** at B3LYP/6–31+g(d) level in methanol.

Configuration	Conformer	E (Hartree)	E (kcal/mol)	Population (%)
R-**8**	1	–846.6892129	–531305.947986879	11.34
R-**8**	2	–846.6893122	–531306.010298622	12.60
R-**8**	3	–846.6883416	–531305.401237416	4.50
R-**8**	4	–846.6888889	–531305.744673639	8.05
R-**8**	5	–846.6877075	–531305.003333325	2.30
R-**8**	6	–846.6884325	–531305.458278075	4.96
R-**8**	7	–846.6893129	–531306.010737879	12.61
R-**8**	8	–846.6877371	–531305.021907621	2.37
R-**8**	9	–846.6892137	–531305.948488887	11.35
R-**8**	10	–846.6877379	–531305.022409629	2.38
R-**8**	11	–846.6877075	–531305.003333325	2.30
R-**8**	12	–846.6888885	–531305.744422635	8.04
R-**8**	13	–846.6877078	–531305.003521578	2.30
R-**8**	14	–846.685133	–531303.38780883	0.15
R-**8**	15	–846.688809	–531305.69453559	7.39
R-**8**	16	–846.6884321	–531305.458027071	4.96
R-**8**	17	–846.6877383	–531305.022660633	2.38
S-**8**	1	–846.6892128	–531305.947924128	10.38
S-**8**	2	–846.6893122	–531306.010298622	11.53
S-**8**	3	–846.6893125	–531306.010486875	11.54
S-**8**	4	–846.6892129	–531305.947986879	10.38
S-**8**	5	–846.6888093	–531305.694723843	6.77
S-**8**	6	–846.6888889	–531305.744673639	7.36
S-**8**	7	–846.6877076	–531305.003396076	2.10
S-**8**	8	–846.6884326	–531305.458340826	4.54
S-**8**	9	–846.6893129	–531306.010737879	11.54
S-**8**	10	–846.6877371	–531305.021907621	2.17
S-**8**	11	–846.6892141	–531305.948739891	10.39
S-**8**	12	–846.6877379	–531305.022409629	2.17
S-**8**	13	–846.6877074	–531305.003270574	2.10
S-**8**	14	–846.6851341	–531303.388499091	0.14
S-**8**	15	–846.6884317	–531305.457776067	4.54

S-**8**	16	–846.687738	–531305.02247238	2.17
S-**8**	17	–846.6852468	–531303.459219468	0.15

R-**8**

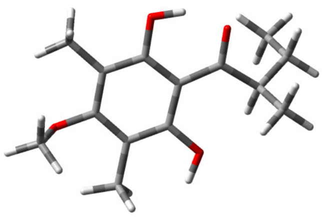

Conf.1 (11.34%)

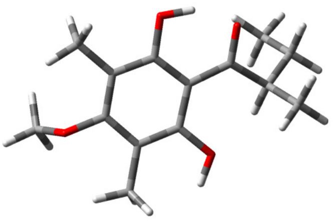

Conf.2 (12.60%)

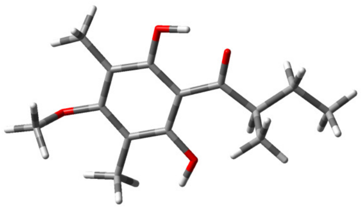

Conf.3 (4.50%)

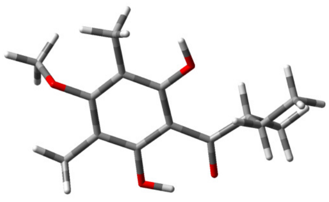

Conf.4 (8.05%)

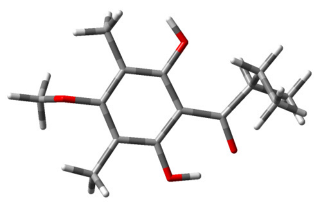

Conf.5 (2.30%)

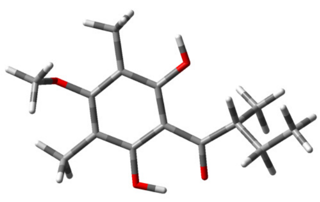

Conf.6 (4.96%)

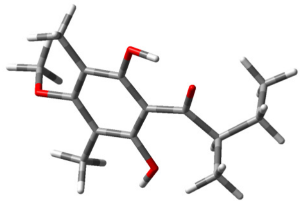

Conf.7 (12.61%)

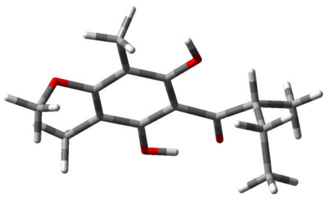

Conf.8 (2.37%)

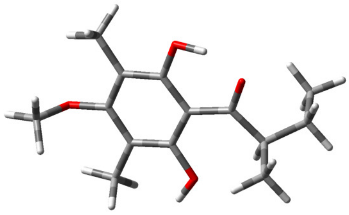

Conf.9 (11.35%)

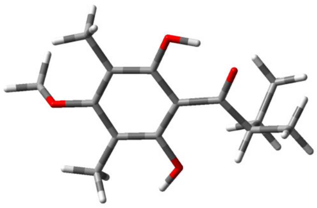

Conf.10 (2.38%)

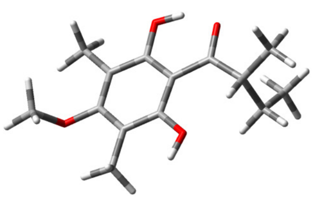

Conf.11 (2.30%)

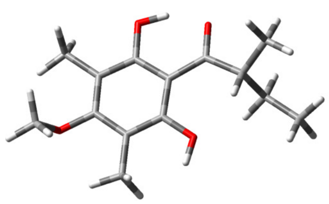

Conf.12 (8.04%)

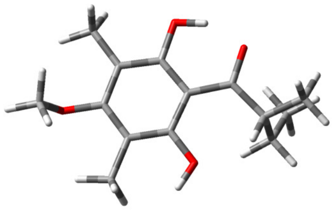

Conf.13 (2.30%)

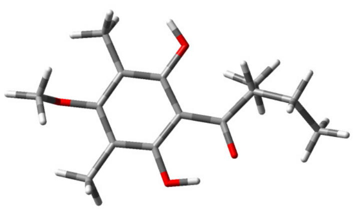

Conf.14 (0.15%)

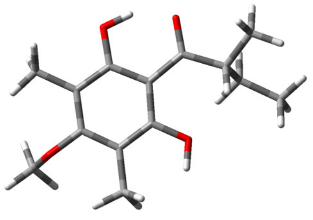

Conf.15 (7.39%)

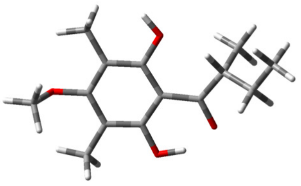

Conf.16 (4.96%)

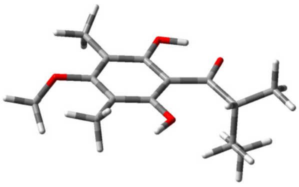

Conf.17 (2.38%)

S-**8**

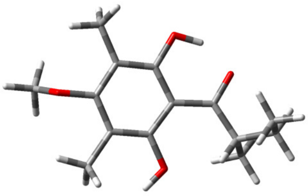

Conf.1 (10.38%)

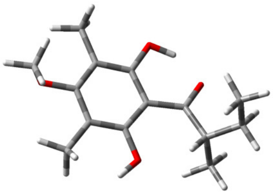

Conf.2 (11.53%)

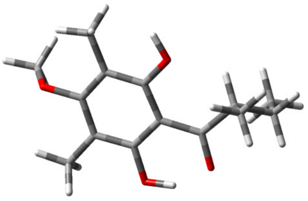

Conf.3 (11.54%)

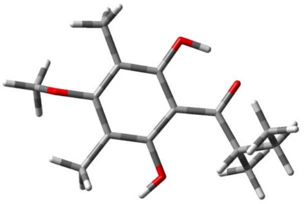

Conf.4 (10.38%)

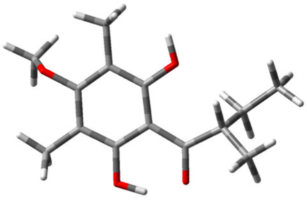

Conf.5 (6.77%)

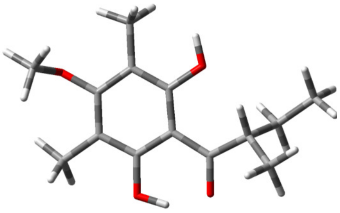

Conf.6 (7.36%)

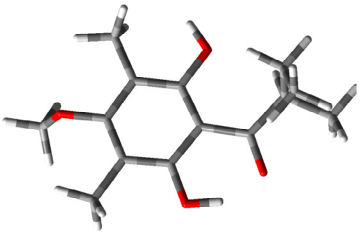

Conf.7 (2.10%)

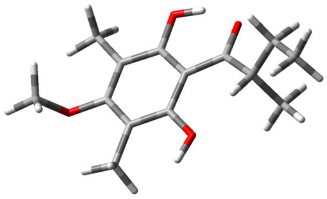

Conf.8 (4.54%)

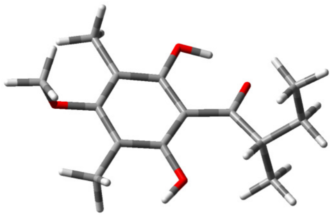

Conf.9 (11.54%)

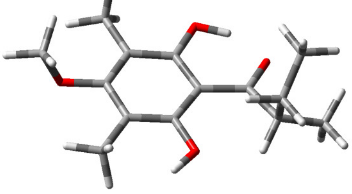

Conf.10 (2.17%)

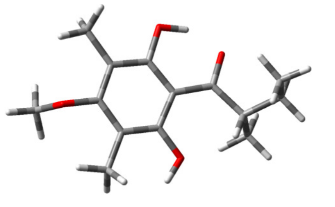

Conf.11 (10.39%)

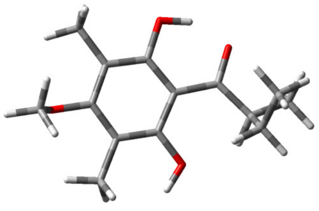

Conf.12 (2.17%)

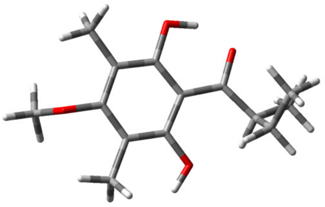

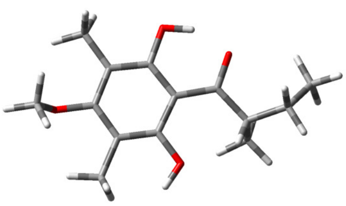

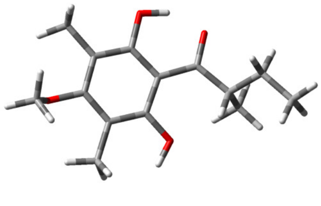

Conf.13 (2.10%) Conf.14 (0.14%) Conf.15 (4.54%)

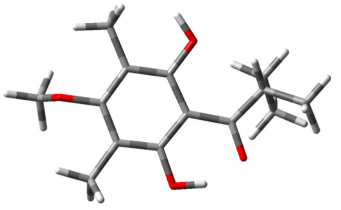

Conf.16 (2.17%)

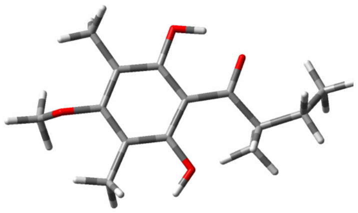

Conf.17 (0.15%)

Figure S20. The optimized conformers and equilibrium populations of **8**.

Physicochemical data of known compounds **2**, **4**–**8.**

Aspergillusether J (**1**). white amorphous solid; UV (MeOH) λ_{max} (logε) 285 (2.40), 207 (3.47) nm; IR (film) ν_{max} 3435, 1683, 1575, 1409, 1338, 1213, 1190, 1070 cm^{-1}; ^{1}H NMR (500 MHz, methanol-d_4): δ_H 6.01 (1H, s, H-1), 5.12 (1H, br s, H-2″), 3.80 (3H, s, 3′-OCH_3), 2.24 (3H, s, 2′-CH_3), 2.20 (3H, 6-CH_3), 1.64 (3H, s, H_3-4″), 1.52 (3H, d, J = 6.5 Hz, H_3-3″); ^{13}C NMR (125 MHz, methanol-d_4) δ_C: 154.7 (C, C-2), 153.5 (C, C-3′), 151.3 (C, C-4), 149.2 (C, C-1′), 138.1 (C, C-6′), 137.2 (C, C-5′), 136.1 (C, C-6), 130.6 (C, C-1″), 127.7 (CH, C-2″), 120.4 (C, C-2′), 118.5 (C, C-4′), 116.2 (C, C-5), 110.2 (C, C-3), 109.4 (CH, C-1), 60.8 (CH_3, 3-OMe), 20.4 (CH_3, 6-CH_3), 16.8 (CH_3, C-4″), 13.6 (CH_3, C-3″), 9.9 (CH_3, 2′-CH_3). HR-ESIMS *m/z* 417.0414 $[M + H]^+$ (calcd for $C_{19}H_{20}Cl_3O_4$, 417.0427), 415.0285 $[M - H]^-$ (calcd for $C_{19}H_{18}Cl_3O_4$, 415.0271).

Aspergillusether F (**2**): white needle crystals; ^{1}H NMR (500 MHz, methanol-d_4): δ_H 6.00 (1H, s, H-1), 5.10 (1H, br s, H-2″), 2.18 (3H, s, 2′-CH_3), 2.17 (3H, s, 6-CH_3), 1.64 (3H, s, H_3-4″), 1.51 (3H, d, J = 6.8 Hz, H_3-3″); ^{13}C NMR (125 MHz, methanol-d_4) δ_C: 155.1 (C, C-2), 151.2 (C, C-4), 150.1 (C, C-3′), 148.8 (C, C-1′), 136.1 (C, C-6), 136.0 (C, C-5′), 134.8 (C, C-6′), 130.8 (C, C-1″), 127.4 (CH, C-2″), 115.9 (C, C-5), 113.7 (C, C-2′), 111.7 (C, C-4′), 110.0 (CH, C-1), 109.4 (C, C-3), 20.4 (CH_3, 6-CH_3), 16.8 (CH_3, C-4″), 13.6 (CH_3, C-3″), 9.7 (CH_3, 2′-CH_3). HR-ESIMS *m/z* 403.0255 $[M + H]^+$ (calcd for $C_{18}H_{18}Cl_3O_4$, 403.0271).

Nornidulin (**4**): colorless solid; ^{1}H NMR (500 MHz, methanol-d_4): δ_H 5.38 (1H, q, J = 6.9 Hz, H-2'), 2.45 (3H, s, 1-CH_3), 2.26 (3H, s, 9-CH_3), 1.95 (3H, s, H_3-4'), 1.82 (3H, d, J = 6.9 Hz, H-3'); ^{13}C NMR (125 MHz, methanol-d_4) δ_C: 163.6 (C, C-11), 158.9 (C, C-3), 155.3 (C, C-4a), 150.7 (C, C-

8), 143.3 (C, C-9a), 143.1 (C, C-1), 141.0 (C, C-5a), 136.1 (C, C-6), 131.1 (C, C-1'), 128.7 (CH, C-2'), 121.5 (C, C-2), 117.9 (C, C-9), 117.6 (C, C-7), 115.6 (C, C-11a), 111.8 (C, C-4), 19.0 (CH_3, 1-CH_3), 17.7 (CH_3, C-4'), 14.2 (CH_3, C-3'), 10.1 (CH_3, 9-CH_3). HR-ESIMS *m/z* 429.0067 $[M + H]^+$ (calcd for $C_{19}H_{16}Cl_3O_5$, 429.0063).

Aspergillusidone B (**5**): colorless solid; ^1H NMR (500 MHz, methanol-d_4): δ_H 6.89 (1H, s, H-2), 5.36 (1H, qq, J = 5.3, 1.9 Hz, H-2'), 3.95 (3H, s, 3-OCH_3), 2.49 (3H, s, 1-CH_3), 2.26 (3H, s, 9-CH_3), 1.94 (3H, s, H-4'), 1.80 (3H, d, J = 6.8 Hz, H-3'); ^{13}C NMR (125 MHz, Methanol-d_4) δ_C: 163.9 (C, C-11), 160.4 (C, C-4a), 160.3 (C, C-3), 150.6 (C, C-8), 145.0 (C, C-1), 143.2 (C, C-9a), 143.2 (C, C-5a), 136.2 (C, C-6), 131.2 (C, C-1'), 128.6 (C, C-2'), 117.9 (C, C-9), 117.6 (C, C-7), 115.4 (C, C-11a), 112.5 (C, C-4), 112.2 (C, C-2), 57.3 (CH_3, 3-OCH_3), 21.7 (CH_3, 1-CH_3), 17.8 (CH_3, C-4'), 14.2 (CH_3, C-3'), 10.2 (CH_3, 9-CH_3). HR-ESIMS *m/z* 409.0620 $[M + H]^+$ (calcd for $C_{20}H_{19}Cl_2O_5$, 409.0610).

Aspergillusidone C (**6**): colorless needles; ^1H NMR (700 MHz, methanol-d_4): δ_H 6.53 (1H, s, H-4), 5.37 (1H, q, J = 6.8 Hz, H-2'), 2.46 (3H, s, 1-CH_3), 2.22 (3H, s, 9-CH_3), 1.95 (3H, s, H_3-4'), 1.90 (3H, d, J =6.8 Hz, H_3-3'); ^{13}C NMR (175 MHz, methanol-d_4) δ_C: 164.3 (C, C-11), 162.8 (C, C-3), 159.1 (C, C-4a), 150.3 (C, C-8), 143.4 (C, C-9a), 143.1 (C, C-5a), 142.7 (C, C-1), 135.8 (C, C-6), 130.8 (C, C-1'), 127.9 (CH, C-2'), 121.1 (C, C-2), 117.8 (C, C-9), 117.2 (C, C-11a), 114.8 (C, C-7), 106.4 (CH, C-4), 18.6 (CH_3, 1-CH_3), 17.4 (CH_3, C-4'), 13.7 (CH_3, C-3'), 10.1 (CH_3, 9-CH_3). HR-ESIMS *m/z* 395.0460 $[M + H]^+$ (calcd for $C_{19}H_{17}Cl_2O_5$, 395.0453).

Guisinol (**7**): yellow oil; ^1H NMR (500 MHz, methanol-d_4): δ_H 6.75 (1H, d, J = 1.8 Hz, H-4'), 6.46 (1H, d, J = 1.8 Hz, H-6'), 5.83 (1H, q, J = 6.9 Hz, H-2'''), 5.28 (1H, q, J = 6.7 Hz, H-2''), 2.15 (3H, s, 3-CH_3), 1.97 (3H, s, 2'-CH_3), 1.96 (3H, s, H_3-4'''), 1.95 (3H, s, H_3-4''), 1.77 (3H, d, J = 6.9 Hz, H_3-3'''), 1.72 (3H, d, J = 6.7 Hz, H_3-3''); ^{13}C NMR (125 MHz, methanol-d_4) δ_C: 170.8 (C, C=O), 160.8 (C, C-4), 157.5 (C, C-2), 157.3 (C, C-1'), 151.2 (C, C-3'), 145.2 (C, C-1''), 144.1 (C, C-1'''), 136.9 (C, C-6), 135.9 (C, C-5'), 123.8 (C, C-2''), 122.9 (C, C-2'''), 116.7 (C, C-2'), 113.8 (C, C-3), 112.9 (C, C-5), 110.7 (C, C-6'), 110.6 (CH, C-4'), 106.7 (C, C-1), 17.5 (CH_3, C-4'') 15.5 (CH_3, C-4'''), 14.3 (CH_3, C-3'''), 13.8 (CH_3, C-3''), 9.5 (CH_3 2'-CH_3), 9.0 (CH_3, 3-CH_3). HR-ESIMS *m/z* 417.1469 $[M + H]^+$ (calcd for $C_{23}H_{26}ClO_5$, 417.1469).

1-(2,6-dihydroxy-4-methoxy-3,5-dimethylphenyl)-2-methylbutan-1-one (**8**): brown oil; ^{1}H NMR (700 MHz, methanol-d_4): δ_H 3.75 (1H, m, J = 7.1 Hz, H-8), 3.68 (3H, s, 4-OCH$_3$), 2.09 (3H, s, H$_3$-13), 2.03 (3H, s, H$_3$-12), 1.76 (1H, m, H-9a), 1.40 (1H, m, H-9b), 1.14 (3H, d, J = 6.8 Hz, H$_3$-11), 0.87 (3H, t, J = 7.6 Hz, H$_3$-10); ^{13}C NMR (175 MHz, methanol-d_4) δ_C: 212.2 (C, C-7), 162.0 (C, C-5), 162.0 (C, C-3), 160.0 (C, C-1), 111.1 (C, C-4), 109.0 (C, C-2), 108.5 (C, C-6), 62.8 (CH$_3$, 4-OMe), 46.5 (CH, C-8), 28.6 (CH$_2$, C-9), 17.6 (CH$_3$, C-11), 12.3 (CH$_3$, C-10), 9.3 (CH$_3$, C-13), 8.2 (CH$_3$, C-12).

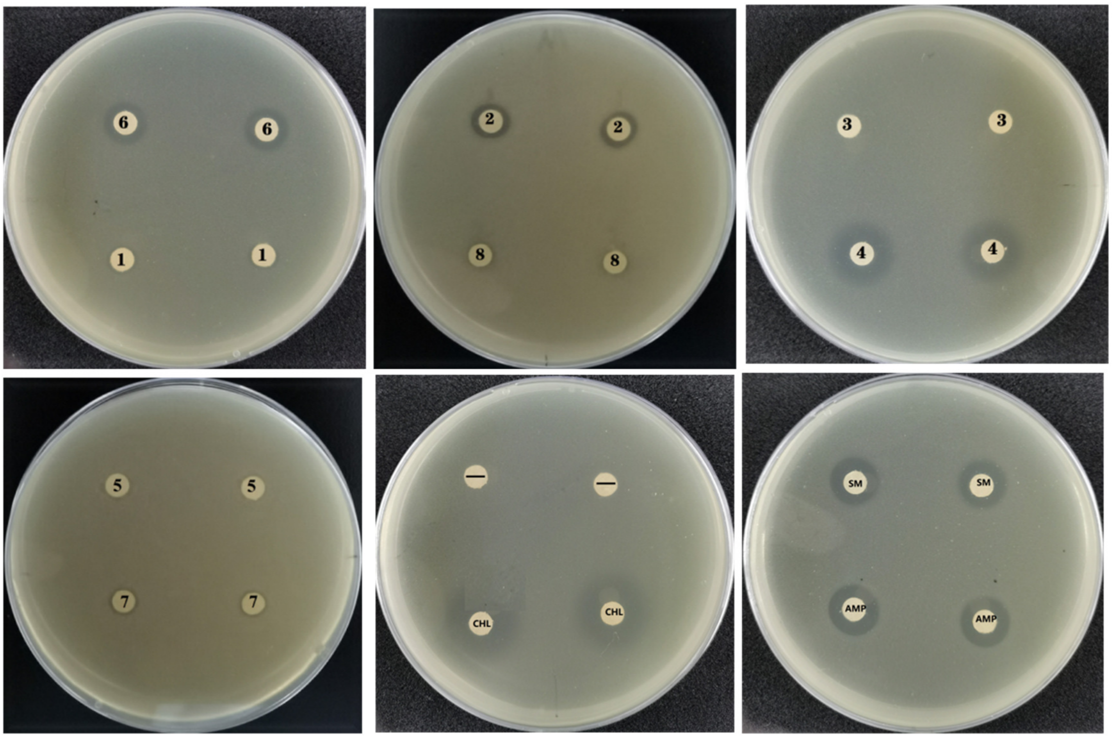

Figure S21. Anti-MRSA activity of compounds **1**–**8** by agar diffusion method. (CHL: Chloramphenicol; SM: Streptomycin; AMP: Ampicillin)

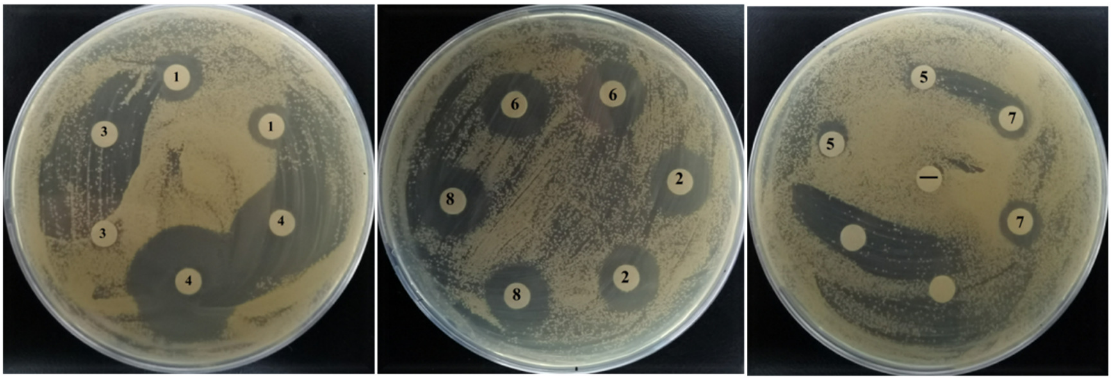

Figure S22. Anti-*M. variabilis* activity of compounds **1**–**8** by agar diffusion method.

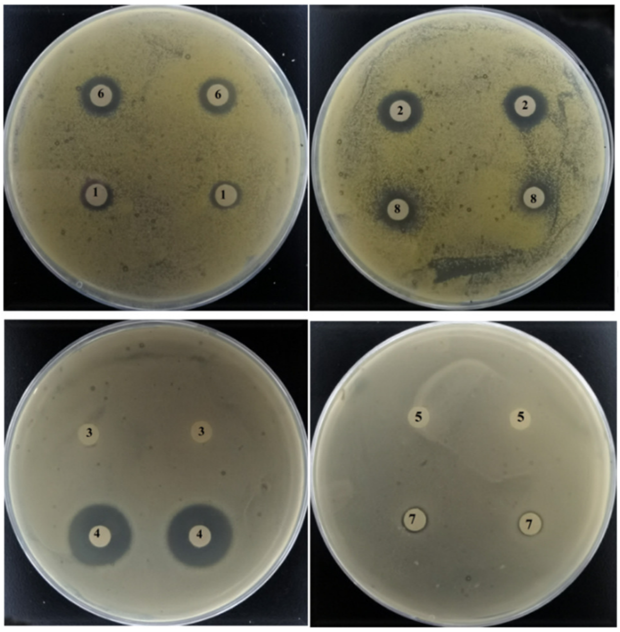

Figure S23. Anti-*M. jannaschii* activity of compounds **1**–**8** by agar diffusion method.

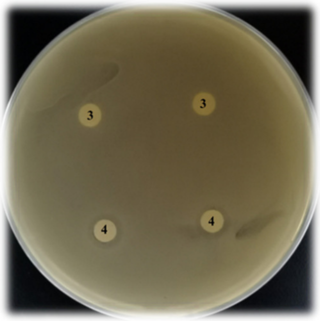

Figure S24. Anti-*V. Pelagius* activity of compound **4** by agar diffusion method.

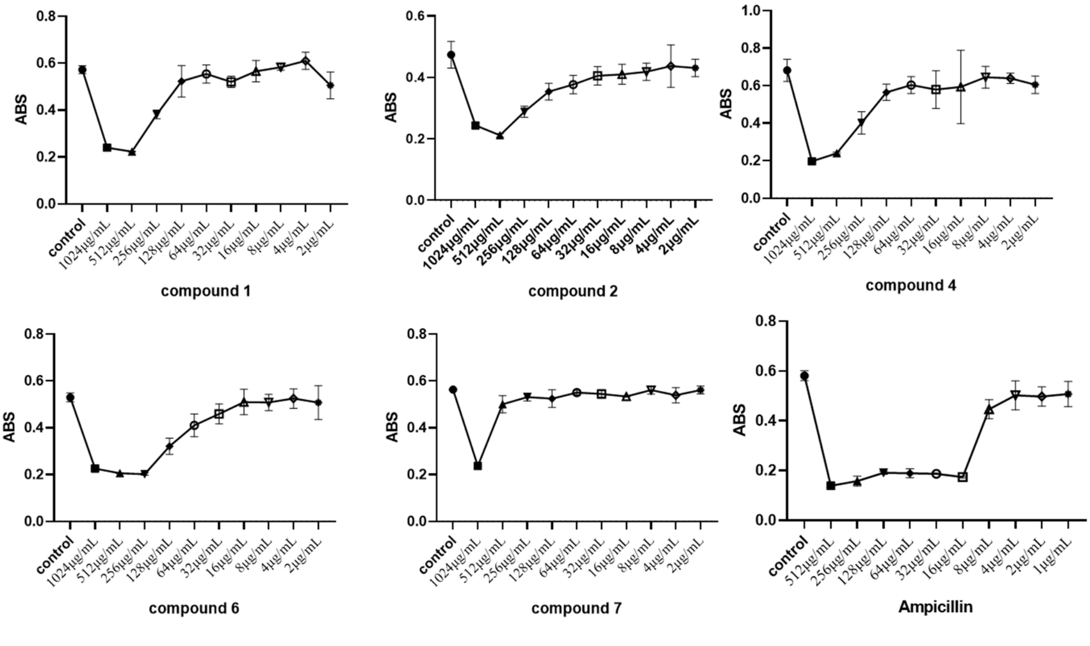

Figure S25. Anti-MRSA activity of compounds **1**, **2**, **4**, **6**, **7** and ampicillin.

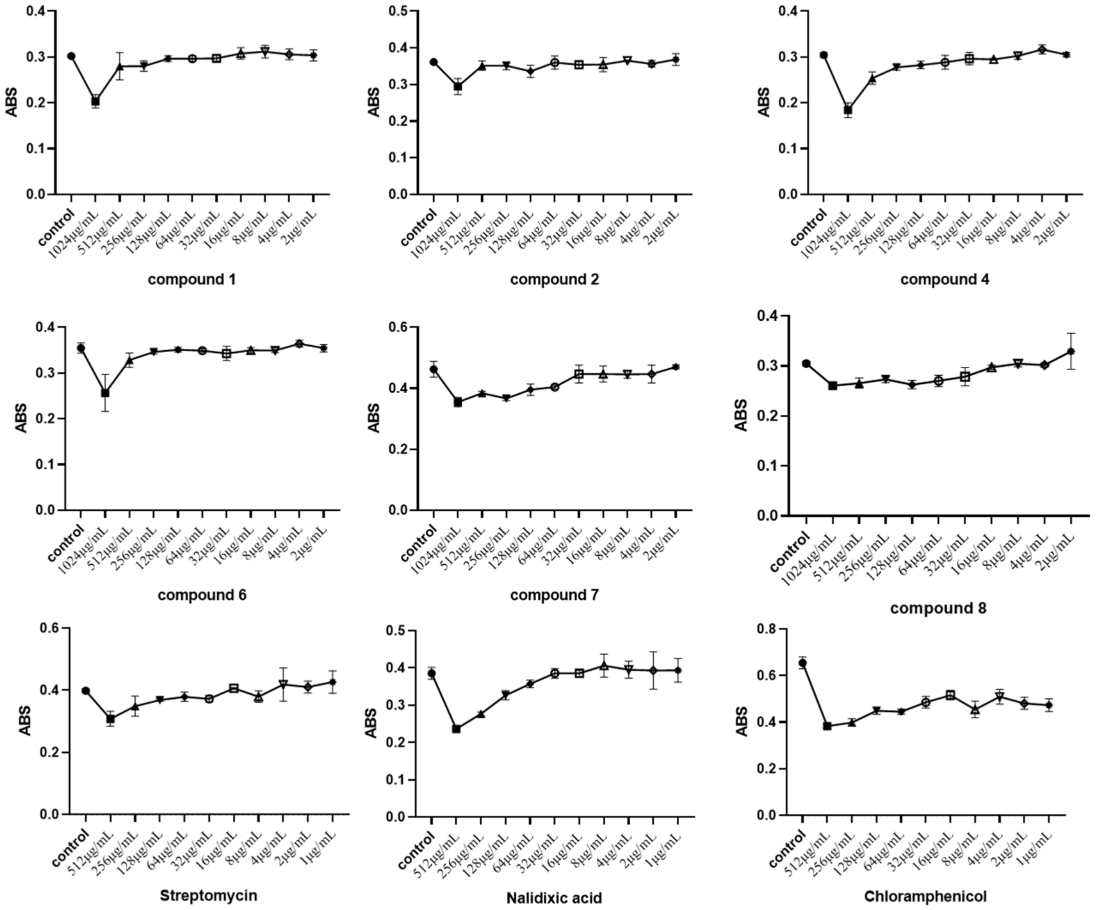

Figure S26. Anti-*M. variabilis* activity of compounds **1**, **2**, **4**, **6~8** and positive control

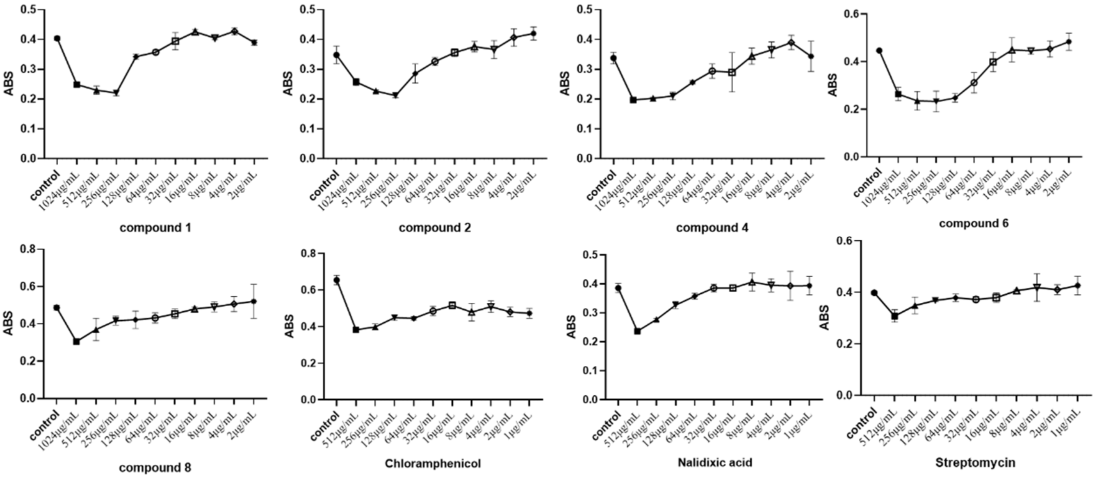

Figure S27. Anti-*M. jannaschii* activity of compounds **1**, **2**, **4**, **6**, **8** and positive control

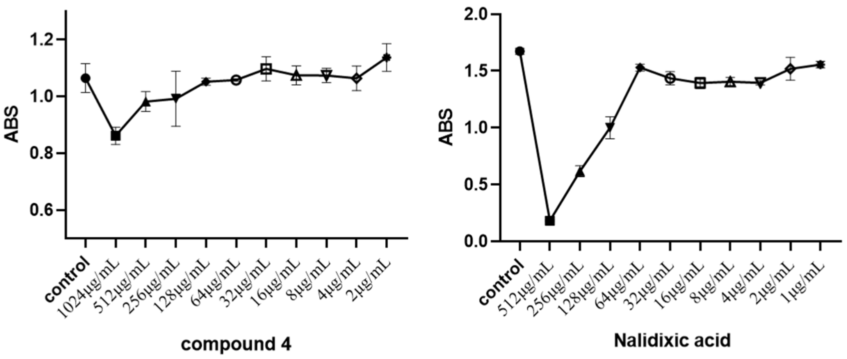

Figure S28. Anti-*V. Pelagius* activity of compounds **4** and nalidixic acid.

The ITS sequence of *Aspergillus unguis* GXIMD 02505

>ITS1

TTTTGAGGAGTCGGCTGCTCGGGCGCCACCTCCCACCCTTGATACTAAACACTGTT

GCTTCGGCGGGGAGCCCCTTCCGGGGGGCAAGCCGCCGGGGACCACTGAACTTC

ATGCCTGAGAGTGATGCAGTCTGAGTCTGAATTATAAATCAGTCAAAACTTTCAAC

AATGGATCTCTTGGTTCCGGCATCGATGAAGAACGCAGCGAACTGCGATAAGTAA

TGTGAATTGCAGAATTCAGTGAATCATCGAGTCTTTGAACGCACATTGCGCCCCCT

GGCATTCCGGGGGGCATGCCTGTCCGAGCGTCATTGCTGCCCTTCAAGCCCGGCT

TGTGTGTTGGGTCGTCGTCCCCCCCGGGGGACGGGCCCGAAAGGCAGCGGCGGC

ACCGTGTCCGGTCCTCGAGCGTATGGGGCTTTGTCACCCGCTCGATTAGGGCCGG

CCGGGCGCCAGCCGGCGTCATCAATCTATTTTACCAGGTTGACCTCGGATCAGGTA

GGGATACCCGCTGAACTTAAGCATATCAATAAGCGGAGGA

>ITS4

ATACTACTGACTACTGATCGAGGTCACCTGGTAATAGATTGATGACGCCGGCTGGC

GCCCGGCCGGCCCTAATCGAGCGGGTGACAAAGCCCCATACGCTCGAGGACCGG

ACACGGTGCCGCCGCTGCCTTTCGGGCCCGTCCCCCGGGGGGGACGACGACCCA

ACACACAAGCCGGGCTTGAAGGGCAGCAATGACGCTCGGACAGGCATGCCCCCC

GGAATGCCAGGGGGCGCAATGTGCGTTCAAAGACTCGATGATTCACTGAATTCTG

CAATTCACATTACTTATCGCAGTTCGCTGCGTTCTTCATCGATGCCGGAACCAAGA

GATCCATTGTTGAAAGTTTTGACTGATTTATAATTCAGACTCAGACTGCATCACTCT

CAGGCATGAAGTTCAGTGGTCCCCGGCGGCTTGCCCCCCGGAAGGGGCTCCCCG

CCGAAGCAACAGTGTTTAGTATTCAAGGGTGGGAGGTTGGGCGCCCGGAGGCAG

CCCGCACTCGGTAATGATCCTTCCGCAGGTTCACCTACGGAAG
